# Hyaluronic acid-bilirubin nanomedicine-based combination chemoimmunotherapy

**DOI:** 10.1038/s41467-023-40270-5

**Published:** 2023-08-08

**Authors:** Yonghyun Lee, Jongyoon Shinn, Cheng Xu, Hannah E. Dobson, Nouri Neamati, James J. Moon

**Affiliations:** 1 Department of Pharmacy, College of Pharmacy, Ewha Womans University, Seoul, 03760 South Korea; 2 Graduate School of Pharmaceutical Sciences, Ewha Womans University, Seoul, 03760 South Korea; 3 Department of Pharmaceutical Sciences, University of Michigan, Ann Arbor, MI 48109 USA; 4 Biointerfaces Institute, University of Michigan, Ann Arbor, MI 48109 USA; 5 Department of Medicinal Chemistry, University of Michigan, Ann Arbor, MI 48109 USA; 6 Department of Biomedical Engineering, University of Michigan, Ann Arbor, MI 48109 USA; 7 Department of Chemical Engineering, University of Michigan, Ann Arbor, MI 48109 USA

**Keywords:** Immunotherapy, Biomedical engineering, Drug delivery

## Abstract

Despite significant advances in immune checkpoint blockade (ICB), immunosuppression mediated by tumor-associated myeloid cells (TAMCs) poses a major barrier to cancer immunotherapy. In addition, while immunogenic cell death (ICD) provides a viable approach to inducing anti-tumor immune response, it remains unknown how to effectively trigger ICD while addressing immunosuppressive TAMCs. Here, we show that SC144, a gp130 inhibitor that blocks the IL-6/gp130/STAT3 pathway, induces ICD of tumor cells and polarizes macrophages to M1-phenotype in vitro. However, as SC144 also induces killing of CD8^+^ T-cells, we sought to deliver SC144 selectively to tumor cells and TAMCs. Toward this goal, we have developed hyaluronic acid-bilirubin nanoparticles (HABN) that accumulate in CD44^hi^ tumor cells and TAMCs. Systemic administration of SC144 loaded in HABN (SC144@HABN) induces apoptosis and ICD of tumor cells, increases the ratio of M1-like to M2-like macrophages, and decreases the frequency of myeloid-derived suppressor cells and CD4^+^ regulatory T-cells, while promoting anti-tumor CD8^+^ T-cells. Moreover, SC144@HABN combined with anti-PD-L1 ICB efficiently eliminates MC38 tumors and ICB-resistant 4T1 tumors. Overall, our work demonstrates a therapeutic strategy based on coordinated ICD induction and TAMC modulation and highlights the potential of combination chemoimmunotherapy.

## Abstract (web summary)

Immunosuppressive tumour immune microenvironments (TME) limit the success of immune checkpoint blockade (ICD). Here, the authors develop a hyaluronic acid-bilirubin nanoparticle (HABN) capable of inducing immunogenic cell death in tumour cells and altering the TME, resulting in increased sensitivity to ICB (anti-PD-L1) in preclinical models of colorectal cancer and breast cancer.

## Introduction

Cancer immunotherapy aims to harnesses the immune system to eliminate cancer^1–4^. Immune checkpoint blockers (ICBs) targeting CTLA-4, PD-1, and PD-L1 have generated unprecedented anti-tumor responses in cancer patients^1–3^. However, only 10–30% of patients currently respond to ICBs^5,6^. The therapeutic efficacy of ICBs is generally thought to depend on pre-existing anti-tumor T-cell immunity. Thus, poor tumor-infiltration of T-cells and immunosuppressive tumor microenvironment (TME) observed in most advanced cancer patients is attributed to the limited patient response rate to ICB therapy^1,2^. Therefore, various strategies, including therapeutic vaccines, radiation therapy, and chemotherapy, are being developed to improve anti-tumor T-cell response and reverse immunosuppression in the TME so that combination immunotherapy with ICBs can promote stronger anti-tumor immunity^7–9^.

Notably, immunogenic cell death (ICD), a special form of tumor-cell killing, induced by certain chemotherapeutic drugs, such as bleomycin, doxorubicin, and oxaliplatin, may contribute to anti-tumor immune response^10,11^. During ICD process, tumor cells expose “eat me”, “danger”, and “find me” signals. Once the “eat me” signals, such as calreticulin (CRT), are exposed on the surfaces of immunogenically dying tumor cells, they promote phagocytosis and presentation of tumor antigens by dendritic cells (DCs) in the context of major histocompatibility complex (MHC) class I or II, triggering antigen-specific T cell responses^12,13^. In addition, danger signals, such as high-mobility group box 1 (HMGB1), released from immunogenically dying tumor cells can activate DCs via interactions with pattern-recognition receptors and facilitate antigen presentation by DCs, leading to enhanced antigen-specific T cell responses^11,14^. In parallel, “find me” signals, such as C-X-C motif chemokine ligand 10 (CXCL10), are released from immunogenically dying tumor cells, promoting intratumoral infiltration of anti-tumor T-cells^15,16^. Thus, ICD-inducing chemotherapeutic agents may provide an effective pathway to kill cancer cells while simultaneously eliciting anti-tumor T cell responses^16–18^. Indeed, clinical studies have shown anti-tumor immune responses induced by ICD-inducing drugs, such as doxorubicin, given either as a monotherapy or combined with immunotherapy^19^. However, there are still concerns of limited intratumoral accumulation of ICD agents as well as their off-target toxicities^7,8,20^. Moreover, ICD agents should be delivered to tumor cells while leaving other cells intact, since ICD agents taken up by immune cells in the TME can interfere with anti-tumor immune functions.

Another barrier in cancer immunotherapy is tumor-associated myeloid cells (TAMCs). TAMCs, such as tumor-associated macrophages (TAMs) and myeloid-derived suppressor cells (MDSCs), are the major immune-suppressive component of TME, as they secrete various immune-regulatory factors, such as IL-6 and TGF-β, and inhibit activation, viability, and tumoral-infiltration of T-cells, thus leading to ICB resistance and poor prognosis^21–23^. Various studies have indicated that TAMC modulators, such as imiquimod, 852A, IMO-2055, and BLZ945, can improve ICB-induced anti-tumor immune response^21^. However, these TAMC modulators administered systemically lead to poor accumulation in TAMCs and cause significant off-target toxicities^7,24^. Hence, to overcome such resistance mechanisms and reduce the potential toxicity of TAMC modulators, a drug delivery approach for targeted delivery to TAMCs is needed.

To address these challenges, we sought to develop a general strategy for delivering an ICD agent and TAMC modulator in a manner compatible with ICB therapy. In particular, we previously reported the development of SC144, a small-molecule gp130 inhibitor that can block IL-6-induced nuclear translocation of STAT3, and shown their anti-tumor efficacy in murine xenograft tumor models^25–28^. STAT3 is an oncogenic transcription factor with potent immunosuppressive functions^29–31^, and activation of the IL-6/gp130/STAT3 pathway is associated with tumor progression and M2-polarized TAMs with pro-tumoral effects^32–34^. Moreover, it has been reported that STAT3 inhibitor can improve ICD of tumor cells^35–37^. Thus, here we examined the impact SC144 on ICD of tumor cells and polarization of macrophages.

**Fig. 1:**
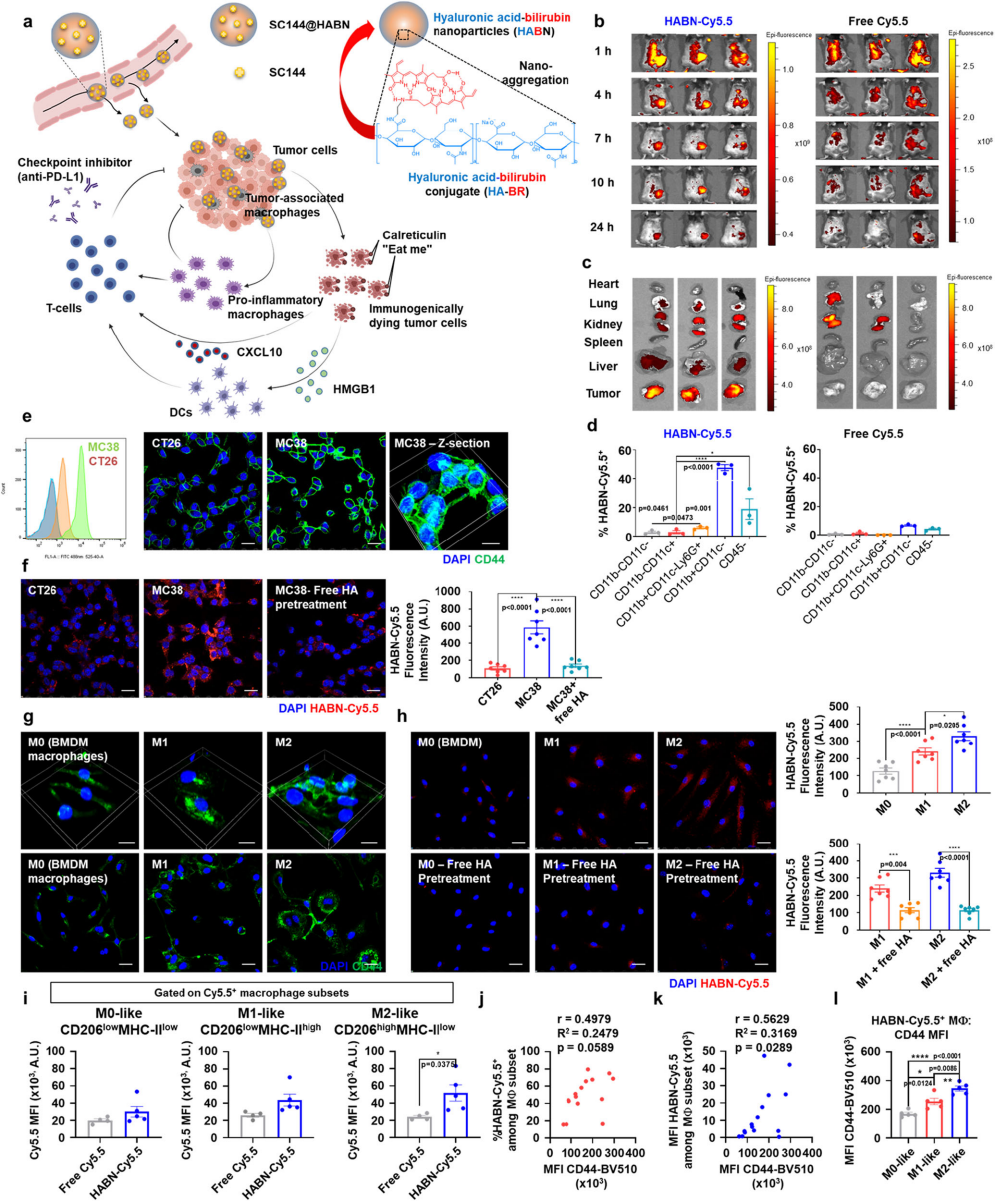
Hyaluronic acid-bilirubin nanomedicine (HABN) localizes in tumor cells and tumor-associated myeloid cells (TAMCs) in MC38 tumor-bearing mice. **a** HABN-mediated chemoimmunotherapy. **b**–**d** MC38 tumor-bearing mice were administered IV on day 25 with 10 mg/kg of HABN-Cy5.5 (equivalent mass of free Cy5.5), or 0.25 mg/kg of free Cy5.5. Whole body fluorescence images (**b**) and organ images (**c**) of mice treated with HABN-Cy5.5 or free Cy5.5 by IVIS. **d** Comparison of uptake levels of HABN-Cy5.5 and free Cy5.5 among various immune cells and cancer cells in tumor tissues by flow cytometry. **e** Flow cytometry analysis and confocal microscopy images of CT26 and MC38 cells stained with anti-CD44 antibody. **f** Confocal microscopy images of MC38 cells incubated with HABN-Cy5.5 (20 µg/ml) for 1 h. For the competition study, MC38 cells were pre-treated for 1 h with free HA (2 mg/ml). **g** Confocal microscopy images of BMDM cells pre-incubated with LPS (100 ng/ml) and IFN-γ (10 ng/ml), IL-4 (20 ng/ml), or control medium for 20 h and stained with anti-CD44 antibody. **h**, Confocal microscopy images and HABN-Cy5.5 fluorescence intensities of BMDM cells pre-incubated with LPS (100 ng/ml) and IFN-γ (10 ng/ml), IL-4 (20 ng/ml), or control medium for 20 h, followed by 1 h treatment with HABN-Cy5.5 (20 µg/ml). For the competition study, BMDM cells were pre-treated for 1 h with free HA (2 mg/ml) before the antibody treatment. DAPI was used for nuclei counter staining. Scale bars = 20 μm. **i** MC38 tumor-bearing mice were administered IV with free Cy5.5 or HABN-Cy5.5, followed by flow cytometric analysis for Cy5.5 MFI among Cy5.5^+^ M0-, M1-, and M2-like macrophage subsets. **J**, **k** Pearson correlation between **j** the frequency of HABN-Cy5.5^+^ cells or **k** HABN-Cy5.5 MFI and CD44 MFI among the total macrophages. **l**, The expression level of CD44 among HABN-Cy5.5^+^ macrophage subsets. The data represent mean ± s.e.m., biological replicates with *n* = 3 (**d**), *n* = 7 (**f**, **g**, **h**), *n* = 4-5 (**i**), *n* = 15 (**j**, **k**), or *n* = 5 (**l**). *p < 0.05, ***p < 0.001, ****p < 0.0001, analyzed by one-way ANOVA with Tukey’s HSD multiple comparison post hoc test (**d**, **f**, **h**, **l**), two-sided Student’s T-test (**i**), or Pearson correlation (**j**, **k**). Source data are provided as a Source data file.

Here, we report our discovery that SC144 induces ICD of tumor cells and polarizes immunosuppressive TAMCs into a less immunosuppressive phenotype. However, as we found that SC144 exhibited cytotoxicity among CD8^+^ T-cells, we sought to target the delivery of SC144 to tumor cells and TAMCs while sparing CD8^+^ T-cells. Toward this goal, we have employed hyaluronic acid-bilirubin nanoparticles (HABN) that we have previously developed for CD44-targeted oral administration^38^. Here, we report that HABN administered intravenously (IV) accumulates in CD44-expressing tumor cells and TAMCs. HABN loaded with SC144 (SC144@HABN) allowed for efficient targeted delivery of SC144 to tumor cells and TAMCs. Importantly, SC144@HABN therapy induced ICD of cancer cells, converted TAMCs into less immunosuppressive phenotype, and increased anti-tumor T-cell response, leading to robust anti-tumor efficacy. Furthermore, SC144@HABN achieved robust synergy with anti-PD-L1 ICB therapy in both MC38 and 4T1 murine tumor models (Fig. 1a). Overall, our work demonstrates the potential of SC144@HABN-based induction of ICD and modulation of TAMCs for combination chemoimmunotherapy.

### HABN accumulates in cancer cells and TAMCs

We synthesized hyaluronic acid-bilirubin (HA-BR) conjugate and confirmed its structure using NMR^38^ (Supplementary Fig. 1a and Supplementary Fig. 2). Whereas bilirubin was insoluble in water, HA-BR conjugates with an average conjugation density of ~4 molecules of bilirubin per each 100 K HA molecule were readily dispersed in aqueous solution. HA-BR conjugates self-assembled into nanoparticles (termed HABN) with 83 ± 6 nm diameter and −19 ± 1 mV surface charge in aqueous buffer solution (Supplementary Fig. 1b–d). Next, we investigated whether HABN can target tumor tissues in vivo. We implanted MC38 colon carcinoma cells into subcutaneous flank in C57BL/6 mice, and after 25 days, MC38 tumor-bearing mice were administered intravenously (IV) with either HABN-Cy5.5 or free Cy5.5 (Supplementary Fig. 3). HABN-Cy5.5 fluorescence signal was detected in tumor tissues within 24 h of administration, whereas free Cy5.5 was not detected in tumor tissues (Fig. 1b). IVIS images of major organs showed more efficient accumulation of HABN in tumor tissues, compared with free Cy5.5 (Fig. 1c and Supplementary Fig. 4a). To examine cell types that internalized HABN, we performed flow cytometric analysis on single cell suspension of the excised tumors (Supplementary Fig. 4b, c). Interestingly, robust HABN-Cy5.5 signal was detected in ~45% of CD45^+^CD11b^+^CD11c^-^ TAMCs (*p* < 0.0001) and 20% of CD45^-^ tumor cells (*p* < 0.05), whereas minimal HABN-Cy5.5 signal was detected among CD45^+^CD11b^-^CD11c^-^ cells, CD45^+^CD11b^-^CD11c^+^ DCs, or CD45^+^CD11b^-^CD11c^-^Ly6G^+^ granulocytes (Fig. 1d). In contrast, free Cy5.5 administered IV was detected in only 4–6% of CD45^+^CD11b^+^CD11c^-^ TAMCs and CD45^-^ tumor cells (Fig. 1d). These results indicated that HABN administered IV was efficiently taken up by TAMCs and tumors cells, thus potentially allowing for targeted drug delivery to these cells.

To corroborate these results, we examined cellular uptake of HABN-Cy5.5 by MC38 cells and bone marrow-derived macrophages (BMDMs) in vitro. As HA is a ligand for CD44^38–41^, we also examined the expression levels of CD44 among MC38 cells and BMDMs. We detected high levels of CD44 expression among MC38 cells, M1-like (induced by LPS and IFN-γ), and M2-like (induced by IL-4) BMDMs (Fig. 1e, g). HABN-Cy5.5 was readily taken up by MC38 cells, M1-like, and M2-like BMDMs (Fig. 1f). Free HA added in the media interfered with cellular uptake of HABN by MC38 cells, M1-like, and M2-like BMDMs (Fig. 1f, h). Notably, CT26 colon carcinoma cells and M0 BMDMs exhibited low expression levels of CD44 and low cellular uptake of HABN (Fig. 1e–h). Notably, silencing the expression of CD44 with anti-CD44 siRNA pretreatment significantly reduced the uptake of HABN in both MC38 cells and BMDMs (Supplementary Fig. 5). These results suggest that HABN interacts with CD44 expressed on tumor cells and macrophages, leading to cellular uptake of HABN.

Next, we examined whether HABN administered IV could accumulate in TAMCs as well as tumor cells in vivo. C57BL/6 mice were inoculated with MC38 cells on day 0 and treated on day 11 by IV administration of either soluble Cy5.5 or HABN-Cy5.5. Flow cytometric analysis of tumor tissues on day 12 showed increased accumulation of HABN-Cy5.5 among CD45^+^CD11b^+^F4/80^+^ TAMCs, compared with soluble Cy5.5, especially among Cy5.5^+^CD206^high^MHCII^low^ M2-like macrophages (Fig. 1i and Supplementary Fig. 6). Among TAMCs, there was a correlation between the cellular uptake of HABN-Cy5.5 and the expression level of CD44 (Fig. 1j, k). Among HABN-Cy5.5^+^ CD45^+^CD11b^+^F4/80^+^ TAMCs, the expression level of CD44 was higher on CD206^high^MHCII^low^ M2-like macrophages, compared with CD206^low^MHCII^low^ M0-like macrophages or CD206^low^MHCII^high^ M1-like macrophages (Fig. 1l). Moreover, we observed significantly higher accumulation of HABN-Cy5.5 among CD45^-^CD44^+^ tumor cells, compared with CD45^-^CD44^-^ tumor cells (Supplementary Fig. 7). Taken together, these results suggest that HABN administered IV is taken up by CD44-expressing tumor cells and TAMCs, in particular CD206^high^MHCII^low^ M2-like macrophages.

### SC144 and SC144@HABN polarize macrophages toward M1 phenotype in vitro

SC144 is a small-molecule inhibitor that can block IL-6/gp130/STAT3 pathway^25–28^. As IL-6/gp130/STAT3 activation is associated with immunosuppressive TAMCs, including tumor-associated macrophages^32–34^, we examined whether SC144 can influence M1/M2 polarization of macrophages in vitro. BMDM cells were treated with SC144 and analyzed by flow cytometry. SC144 treatment increased the frequency of CD206^low^MHCII^high^ M1-like macrophages while decreasing CD206^high^MHCII^low^ M2-like macrophages even in the presence of IL-4 that induces M2-like macrophages, compared with PBS (Supplementary Fig. 8), demonstrating SC144-mediated polarization of macrophages toward M1 phenotype.

Next, since HABN accumulates in M2-like TAMCs and tumor cells in vivo (Fig. 1) and SC144 promotes M1 polarization of macrophages (Supplementary Fig. 8), we sought to develop SC144-loaded HABN (termed SC144@HABN). To load SC144 into HABN, we used o/w emulsion with the ultra-sonication method, followed by removing of unloaded drug by dialysis. The resulting SC144@HABN exhibited 144 ± 23 nm diameter and −31 ± 6 mV surface charge (Supplementary Fig. 1d). SC144 was efficiently loaded into HABN with 49.6 ± 1.0% encapsulation efficiency and 9.0% ± 0.2% wt/wt drug loading, as measured by HPLC (Supplementary Fig. 9). When BMDM cells were treated with SC144@HABN, we observed significantly increased the frequency of CD206^low^MHCII^high^ M1-like macrophages (*p* < 0.0001) and decreased frequency of CD206^high^MHCII^low^ M2-like macrophages (*p* < 0.0001), compared with PBS (Supplementary Fig. 8). Moreover, analysis of extracellular cytokine levels showed that SC144@HABN increased the secretion of IL-6 (*p* < 0.0001) and TNF-α (*p* < 0.001) from BMDMs but reduced the immunosuppressive factors, including IL-10 (*p* < 0.0001), TGF-β (*p* < 0.001), and IL-1β (*p* < 0.05) (Supplementary Fig. 10). Notably, M2-like macrophages, but not M0- or M1-like macrophages, exhibited cytotoxicity when treated with either SC144 (*p* < 0.01) or SC144@HABN (*p* < 0.001) (Supplementary Fig. 11). Taken together, these results show that SC144 and SC144@HABN can polarize macrophages toward M1 phenotype in vitro.

### SC144@HABN modulates TAMCs into a less immunosuppressive phenotype in vivo

**Fig. 2:**
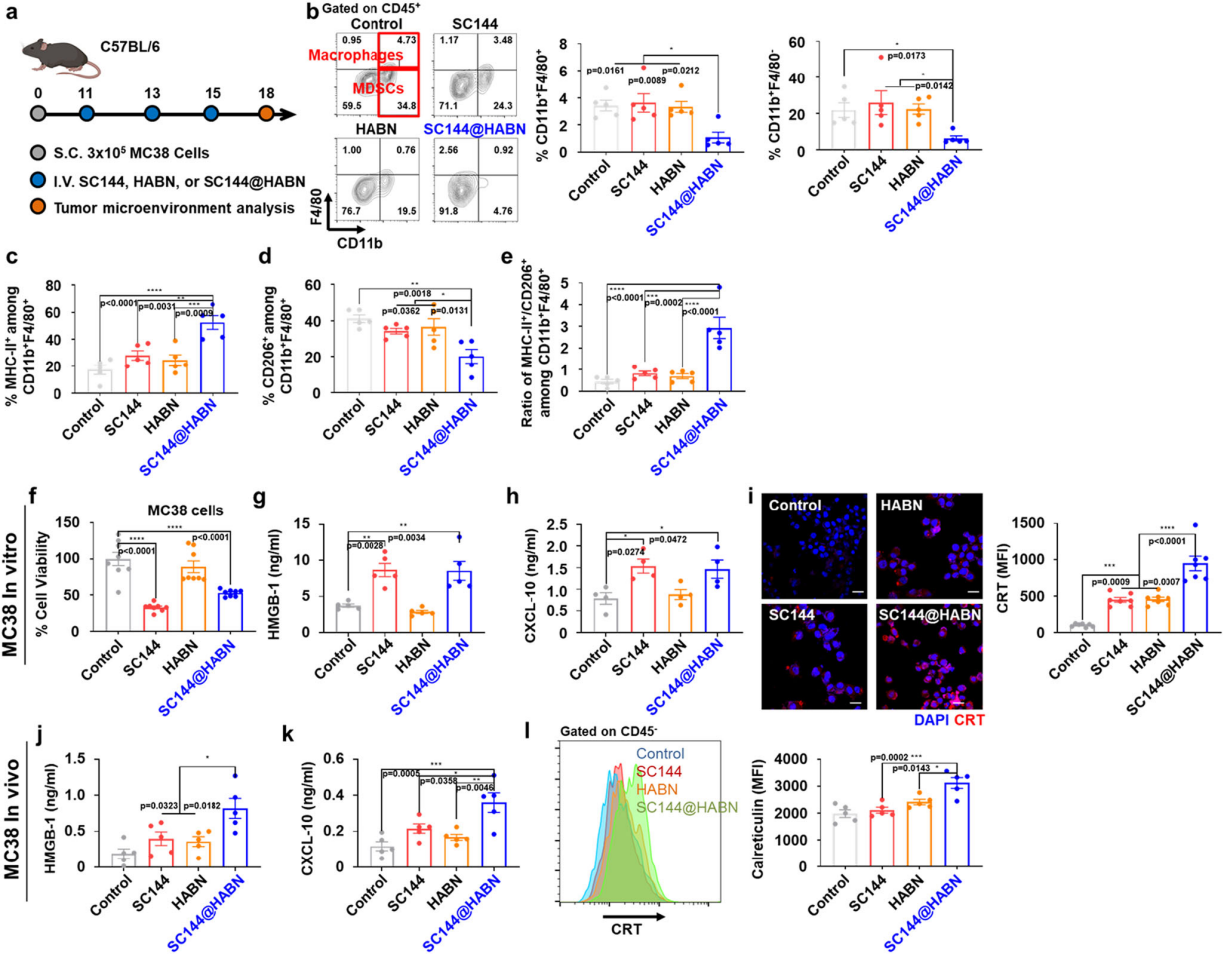
SC144@HABN induces immunogenic cell death of cancer cells and modulates TAMCs. **a**–**e** C57BL/6 mice bearing MC38 tumor were administered IV with SC144 (5 mg/kg), HABN (50 mg/kg) SC144@HABN (5 mg/kg of SC144; 50 mg/kg of HABN), or PBS on days 11, 13, and 15. For the TME analysis, tumor was excised on day 18 and flow cytometry was performed. **b**–**d** Shown are the frequencies of CD45^+^CD11b^+^F4/80^+^ TAMs (**b**, left panel), CD45^+^CD11b^+^ F4/80^-^ MDSCs (**b**, right panel), CD45^+^CD11b^+^F4/80^+^F4/80^+^MHC^+^ M1-like macrophages (**c**), and CD45^+^CD11b^+^F4/80^+^CD206^+^ M2-like macrophages (**d**). **e** The ratio between M1-like and M3-like macrophages. **f** Viability of MC38 cells measured with CCK-8 assay after 24 h treatment with 10 μM of SC144, 40 μg/ml of HABN, SC144@HABN (10 μM of SC144; 40 μg/ml of HABN), or PBS. **g**, **h** HMGB-1 (**g**) and CXCL-10 (**h**) levels released from MC38 cells measured by ELISA after 24 h treatment with 10 μM of SC144, 40 μg/ml of HABN, SC144@HABN (10 μM of SC144; 40 μg/ml of HABN), or PBS. **i** Confocal microscopy images and CRT fluorescence intensity of MC38 cells pre-treated with SC144 (10 μM), HABN (40 μg/ml) or SC144@HABN (10 μM of SC144; 40 μg/ml of HABN), or control medium for 20 h and stained with anti-CRT antibody. **j**–**l** C57BL/6 mice bearing MC38 tumor were administered IV with SC144 (5 mg/kg), HABN (50 mg/kg) SC144@HABN (5 mg/kg of SC144; 50 mg/kg of HABN), or PBS on days 11, 13, and day 15. HMGB-1 (**j**) and CXCL-10 (**k**) plasma levels measured by ELISA. Scale bar = 20 μm. **l** Frequencies of CD45^-^CRT^+^ tumor cells were measured by flow cytometry. The data represent mean ± s.e.m., biological replicates with *n* = 5. *p < 0.05, **p < 0.01, ***p < 0.001, ****p < 0.0001, analyzed by one-way ANOVA with Tukey’s HSD multiple comparison post hoc test. Source data are provided as a Source data file.

Next, we analyzed the impact of SC144@HABN treatment on TAMCs, including tumor-associated macrophages and MDSCs. C57BL/6 mice were inoculated with MC38 cells on day 0 and treated on days 11, 13, and 15 by IV administration of either SC144, HABN, or SC144@HABN (Fig. 2a). Flow cytometric analysis of tumor tissues on day 18 showed that SC144@HABN significantly decreased the frequency of CD11b^+^F4/80^+^ macrophages and CD11b^+^F4/80^-^ MDSCs, compared with PBS (*p* < 0.05, Fig. 2b and Supplementary Fig. 12). In contrast, SC144 or HABN treatment had minimal effects on CD11b^+^F4/80^+^ macrophages and CD11b^+^F4/80^-^ MDSCs, probably due to limited delivery of SC144 to these cell types upon IV administration (Fig. 1). In addition, SC144@HABN treatment significantly increased the frequency of CD11b^+^F4/80^+^MCH-II^+^ M1-like macrophages and decreased the frequency of CD11b^+^F4/80^+^CD206^+^ M2-like macrophages (*p* < 0.01, Fig. 2c, d), leading to a 3.5-fold increase in the ratio of M1-like to M2-like macrophages, compared with SC144 treatment (*p* < 0.001, Fig. 2e).

### SC144@HABN triggers ICD and protects CD8 + T-cells

Next, we examined the impact of SC144 and SC144@HABN on tumor cell killing and ICD. Both SC144 and SC144@HABN induced apoptosis-mediated killing of MC38 cells in vitro, whereas only SC144@HABN IV treatment efficiently induced apoptosis of MC38 tumor in vivo (Fig. 2f, Supplementary Figs. 13, 14), potentially due to increased accumulation of SC144@HABN in tumor tissues (Fig. 1c, Supplementary Fig. 4a). Importantly, SC144 and SC144@HABN promoted robust secretion of HMGB-1 and CXCL-10 from MC38 cells (Fig. 2g, h) and induced the upregulation of calreticulin (CRT) on MC-38 cells (Fig. 2i, Supplementary Fig. 15). Notably, SC144@HABN-mediated upregulation of CRT was lost when MC38 cells were pretreated with anti-CD44 siRNA (Supplementary Fig. 15). As HMGB-1, CXCL-10, and CRT are major markers of ICD, these results suggest that both SC144 and SC144@HABN induce ICD. To corroborate these results in vivo, we treated MC38 tumor-bearing mice with SC144 or SC144@HABN and examined tumor tissues for ICD markers (Fig. 2a). Compared with SC144 treatment, SC144@HABN treatment significantly elevated the serum concentrations of HMGB-1 and CXCL-10 (*p* < 0.05, Fig. 2j, k) and increased CRT levels in CD45^-^ tumor cells (*p* < 0.001, Fig. 2l).

Notably, SC144 was toxic to activated CD8^+^ T-cells over 2 days of culture, but HABN and SC144@HABN exhibited minimal cytotoxicity among CD8^+^ T-cells (Supplementary Fig. 16). This may be attributed to limited uptake of nanoparticles by CD8^+^ T-cells. We observed minimal (<1%) uptake of CD8^+^ HABN-Cy5.5 among either splenocytes, freshly isolated CD8^+^ T-cells, or CD3/CD28-activated CD8^+^ T-cells, whereas free Cy5.5 dye was associated with >95% of CD8^+^ T-cells within 1 h of incubation (Supplementary Fig. 17). While the CD44 activation status of CD8^+^ T-cells largely did not affect the uptake of HABN-Cy5.5, we observed slightly higher uptake of HABN-Cy5.5 among CD3/CD28-activated CD44^hi^CD8^+^ T-cells (Supplementary Fig. 17a). Taken together, these results show that free SC144 treatment promotes ICD of cancer cells but also induces cytotoxicity in CD8 + T-cells. In contrast, HABN-mediated delivery of SC144 enhances ICD while protecting CD8^+^ T-cells against SC144-induced cytotoxicity, potentially due to the cytoprotective activity of HABN as we have reported previously^38^. Similarly, SC144@HABN also protected HepG2 cells, a hepatocyte cell line with low CD44 expression, from SC144-associated cytotoxicity (Supplementary Fig. 18).

### SC144@HABN activates CD8 + T-cell response and exerts anti-tumor efficacy in vivo

**Fig. 3:**
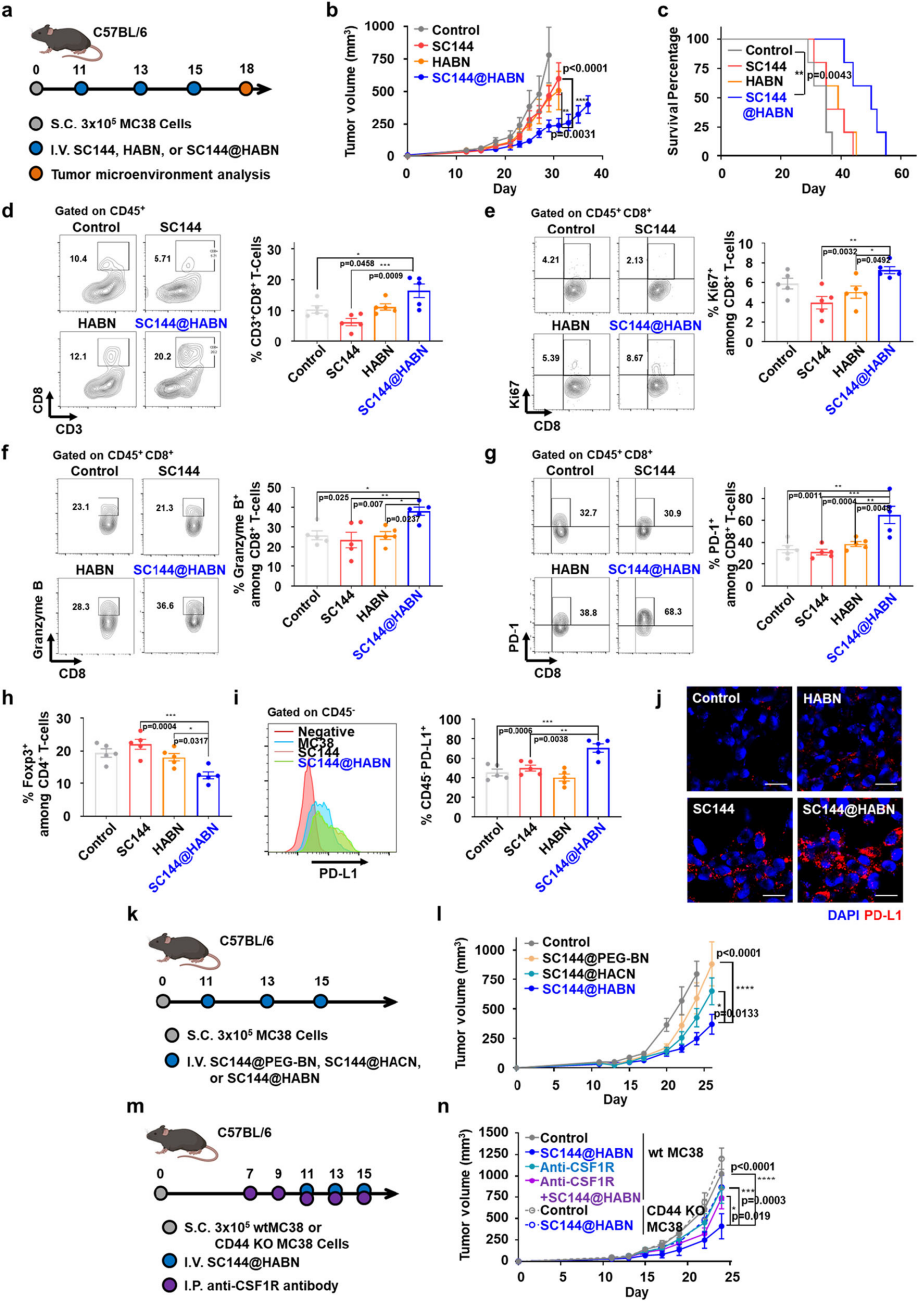
SC144@HABN improves anti-cancer activity of SC144. **a**–**i** C57BL/6 mice bearing MC38 tumor were administered IV with SC144 (5 mg/kg), HABN (50 mg/kg), SC144@HABN (5 mg/kg of SC144; 50 mg/kg of HABN), or PBS on days 11, 13, and day 15. For TME analysis, tumor was excised on day 18 and flow cytometry was performed. **b** MC38 tumor growth curves and **c**, survival of mice treated with the indicated formulations. **d**–**i**, Shown are the frequencies of intratumoral CD45^+^CD3^+^CD8^+^ T-cells (**d**), CD45^+^CD3^+^CD8^+^Ki67^+^ T-cells (**e**), CD45^+^CD3^+^CD8^+^Granzyme B^+^ T-cells (**f**), CD45^+^CD3^+^CD8^+^PD-1^+^ T-cells (**g**), CD45^+^CD3^+^CD4^+^Foxp3^+^ T-cells (**h**), and CD45^-^PD-L1^+^ cells (**i**). **j** Confocal microscopy images of MC38 cells pre-treated with SC144 (10 μM), HABN (40 μg/ml) or SC144@HABN (10 μM of SC144; 40 μg/ml of HABN), or control medium for 20 h and stained with anti-PD-L1 antibody. Scale bar = 20 μm. **k**, **l** Tumor growth curves of C57BL/6 mice bearing MC38 tumor administered IV with PBS, SC144@PEG-BN, SC144@HACN, or SC144@HABN (5 mg/kg of SC144 and 50 mg/kg of either PEG-BN, HACN, or HABN) on days 11, 13, and 15. **m**, **n** Tumor growth curves of C57BL/6 mice inoculated with MC38 or CD44-KO MC38 tumor cells and administered IV with SC144@HABN (5 mg/kg of SC144; 50 mg/kg of HABN) or PBS on days 11, 13, and 15. For macrophage depletion, 20 mg/kg of anti-CSF1R antibody were administered i.p. on days 7, 9, 11, 13, and 15. The data represent mean ± s.e.m., biological replicates with *n* = 5. *p < 0.05, **p < 0.01, ***p < 0.001, and ****p < 0.0001, analyzed by two-way ANOVA (**b**, **l**, **n**), or one-way ANOVA (**d**–**i**) with Tukey’s HSD multiple comparison post hoc test, or Kaplan–Meier survival analysis with the log-rank (Mantel–Cox) test (**c**). Source data are provided as a Source data file.

We evaluated the therapeutic efficacy of SC144@HABN in vivo. Mice bearing MC38 tumor were treated with SC144, HABN, or SC144@HABN on days 11, 13, and 15 and monitored for tumor growth (Fig. 3a). SC144@HABN treatment significantly decreased tumor growth, compared with free SC144 treatment (*p* < 0.0001, Fig. 3b), and extended animal survival (Fig. 3c). To understand the impact of SC144@HABN on CD8^+^ T-cells in the TME, treated MC38 tumors were analyzed on day 18 (Fig. 3a and Supplementary Fig. 12). SC144@HABN treatments significantly improved tumor infiltration of CD8^+^ T-cells, compared with SC144 treatment (*p* < 0.01, Fig. 3d). SC144@HABN treatment also induced proliferation (Ki67^+^) and activation (granzyme B^+^) of tumor-infiltrating CD8^+^ T-cells, as compared with SC144 treatment (*p* < 0.01, Fig. 3e, f). SC144@HABN also significantly increased the frequencies of PD-1^+^CD8^+^ T-cells (*p* < 0.001, Fig. 3g), while decreasing the frequencies of CD4^+^FoxP3^+^ regulatory T-cells in the TME (*p* < 0.001, Fig. 3h), as compared with SC144 treatment. Notably, SC144@HABN-treated tumors had a significantly higher frequency of CD45^-^PD-L1^+^ tumor cells in the TME (*p* < 0.01, Fig. 3i), compared with SC144-treated tumors. In line with this result, MC38 cells treated with SC144@HABN in vitro increased the surface expression of PD-L1 on MC38 cells, as shown by confocal microscopy (Fig. 3j). Notably, SC144 treatment also induced PD-L1 expression on MC38 cells in vitro (Fig. 3j) but failed to increase PD-L1 expression on MC38 cells in vivo (Fig. 3i), potentially due to limited tumor accumulation upon IV administration (Fig. 1b–d). These results indicated that SC144@HABN exerted robust anti-tumor efficacy in vivo and that SC144@HABN activated CD8^+^ T-cells and increased the expression of PD-L1 among tumor cells.

Next, we sought to understand what factors are important for the therapeutic efficacy of SC144@HABN. We first examined the effect of HA and BN on the anti-tumor efficacy of SC144@HABN. We compared the therapeutic efficacy of SC144@HABN with that of SC144 encapsulated into either PEGylated bilirubin nanoparticles (SC144@PEG-BN) or hyaluronic acid-cholesterol nanoparticles (SC144@HACN). The therapeutic efficacy of SC144@PEG-BN and SC144@HACN was significantly lower than that of SC144@HABN (Fig. 3k, l, Supplementary Fig. 19), indicating the crucial role of both BR and HA in the anti-tumor efficacy of SC144@HABN. Next, we examined the role of macrophages during SC144@HACN treatment by administering IgG antibody against colony-stimulating factor 1 receptor (anti-CSF1R) known to deplete macrophages^42^. Treatment with anti-CSF1R IgG significantly decreased the anti-tumor efficacy of SC144@HABN (Fig. 3m, n). Moreover, we observed SC144@HABN treatment lost its anti-tumor efficacy in mice bearing CD44-KO MC38 tumor cells pre-treated in vitro with CRISPR/Cas9 to knock-down the CD44 expression (Fig. 3m, n, Supplementary Fig. 20). Taken together, these results show the importance of HABN-mediated delivery of SC144, modulation of macrophages, and CD44 expression among tumor cells for the observed anti-tumor efficacy of SC144@HABN.

### SC144@HABN and anti-PD-L1 combo elicits strong anti-tumor immunity

**Fig. 4:**
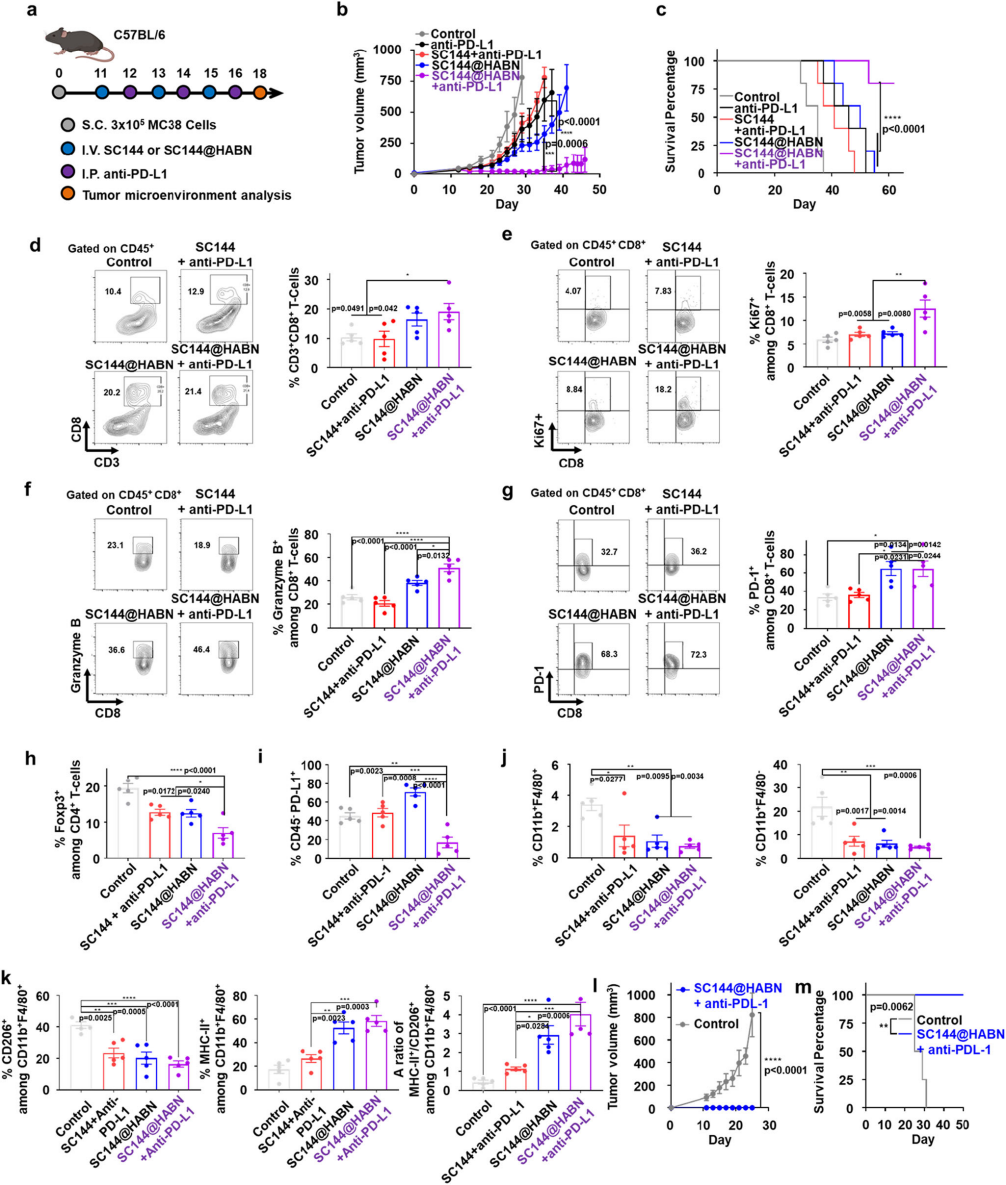
SC144@HABN and anti-PD-L1 combo therapy induces robust anti-tumor immunity. **a**–**k** C57BL/6 mice bearing MC38 tumor were administered IV with SC144 (5 mg/kg), HABN (50 mg/kg) SC144@HABN (5 mg/kg of SC144; 50 mg/kg of HABN), or PBS on days 11, 13, and day 15 with or without intraperitoneal administration of anti-mouse PD-L1 (5 mg/kg) on days 12, 14, and 16. For TME analysis, tumor was excised on day 18, and flow cytometry was performed. **b** MC38 tumor growth curves and **c**, survival of mice treated with the indicated formulations. **d**–**j** Shown are the frequencies of intratumoral CD45^+^CD3^+^CD8^+^ T-cells (**d**), CD45^+^CD3^+^CD8^+^Ki67^+^ T-cells (**e**), CD45^+^CD3^+^CD8^+^Granzyme B^+^ T-cells (**f**), CD45^+^CD3^+^CD8^+^PD-1^+^ T-cells (**g**), CD45^+^CD3^+^CD4^+^Foxp3^+^ T-cells (**h**), CD45^-^PD-L1^+^ cells (**i**), CD45^+^CD11b^+^F4/80^+^ TAMs (**j**, left panel), and CD45^+^CD11b^+^ F4/80^-^ MDSCs (**j**, right panel). **k** Shown are the frequency of CD45^+^CD11b^+^F4/80^+^MHC^+^ M1-like macrophages, CD45^+^CD11b^+^F4/80^+^ CD206^+^ M2-like macrophages, and their ratio. **l**, **m** Tumor-free survivors were inoculated with MC38 cells and tumor volume (**l**) and survival percentages (**m**) were measured. The data represent mean ± s.e.m., biological replicates with *n* = 4 (**l**, **m**) or 5 (**a**–**k**). *p < 0.05, **p < 0.01, ***p < 0.001, ****p < 0.0001, analyzed by two-way ANOVA (**b**, **l**, **m**), or one-way ANOVA (**d**–**k**) with Tukey’s HSD multiple comparison post hoc test, or Kaplan–Meier survival analysis with the log-rank (Mantel–Cox) test (**c**). Source data are provided as a Source data file.

Based on SC144@HABN-mediated upregulation of PD-L1 on tumor cells (Fig. 3I, j), we sought to improve the anti-tumor efficacy of SC144@HABN by co-administration of anti-PD-L1 antibody. MC38 tumor-bearing mice were treated as in Fig. 3a with the addition of anti-PD-L1 antibody administered intraperitoneally after one day of each SC144-based therapy (Fig. 4a).

SC144@HABN therapy combined with anti-PD-L1 antibody exerted robust anti-tumor efficacy and significantly decreased tumor growth, compared with SC144@HABN or SC144 + anti-PD-L1 therapy (*p* < 0.001 and *p* < 0.0001, respectively, Fig. 4b). SC144@HABN + anti-PD-L1 also significantly extended the animal survival with complete response in ~80% animals (*p* < 0.0001, Fig. 4c), whereas animals treated with anti-PD-L1 alone or SC144 + anti-PD-L1 therapy showed minimal survival benefit. To understand the impact of SC144@HABN + anti-PD-L1 combo therapy on the TME, we analyzed tumor-infiltrating lymphocytes on day 18. Compared with free SC144 + anti-PD-L1 combo, SC144@HABN + anti-PD-L1 combo therapy significantly increased the frequency of tumor-infiltrating CD8^+^ T-cells (*p* < 0.05, Fig. 4d) and the surface markers of T-cell proliferation, activation, and exhaustion, as shown by Ki67, granzyme B, and PD-1 staining among CD8^+^ T-cells (Fig. 4e–g). Moreover, SC144@HABN + anti-PD-L1 combo therapy significantly decreased the frequency of CD4^+^FoxP3^+^ regulatory T-cells (Fig. 4h) and CD45^-^PD-L1^+^ tumor cells in the TME (Fig. 4i), compared with other groups. Furthermore, SC144@HABN + anti-PD-L1 combo therapy significantly decreased the frequencies of CD11b^+^F4/80^+^ macrophages and CD11b^+^F4/80^-^ MDSCs (Fig. 4j) and increased the ratio of M1-like to M2-like macrophages by 3.5-fold and 9.1-fold, compared with SC144 + anti-PD-L1 therapy and PBS control (*p* < 0.001, *p* < 0.0001, respectively, Fig. 4k). Importantly, when the survivors from the SC144@HABN + anti-PD-L1 combo group were re-challenged on day 60 with MC38 tumor cells in the contralateral flank, 100% of mice resisted tumor re-challenge (Fig. 4l, m). Furthermore, since SC144 is a gp130 inhibitor and IL-6/gp130 axis is important in both polarization of TAMs and cancer cell growth^25–28,32–34^, we investigated whether IL-6 plays an important role in the anti-tumor efficacy of SC144@HABN + anti-PD-L1 combo therapy. Indeed, the anti-tumor efficacy of SC144@HABN + anti-PD-L1 combo therapy was abrogated by co-treatment with anti-IL-6 antibody, (Supplementary Fig. 21), highlighting an important role of IL-6 modulated by SC144@HABN + anti-PD-L1 combo therapy. Overall, these results show that SC144@HABN + anti-PD-L1 combo therapy increased the frequency of tumor-infiltrating CD8^+^ T-cells and the ratio of M1/M2 macrophages in the TME while decreasing CD4+ Tregs, leading to robust anti-tumor efficacy and establishment of long-term anti-tumor immunity.

### SC144@HABN and anti-PD-L1 combo exerts robust efficacy in 4T1 tumor model

**Fig. 5:**
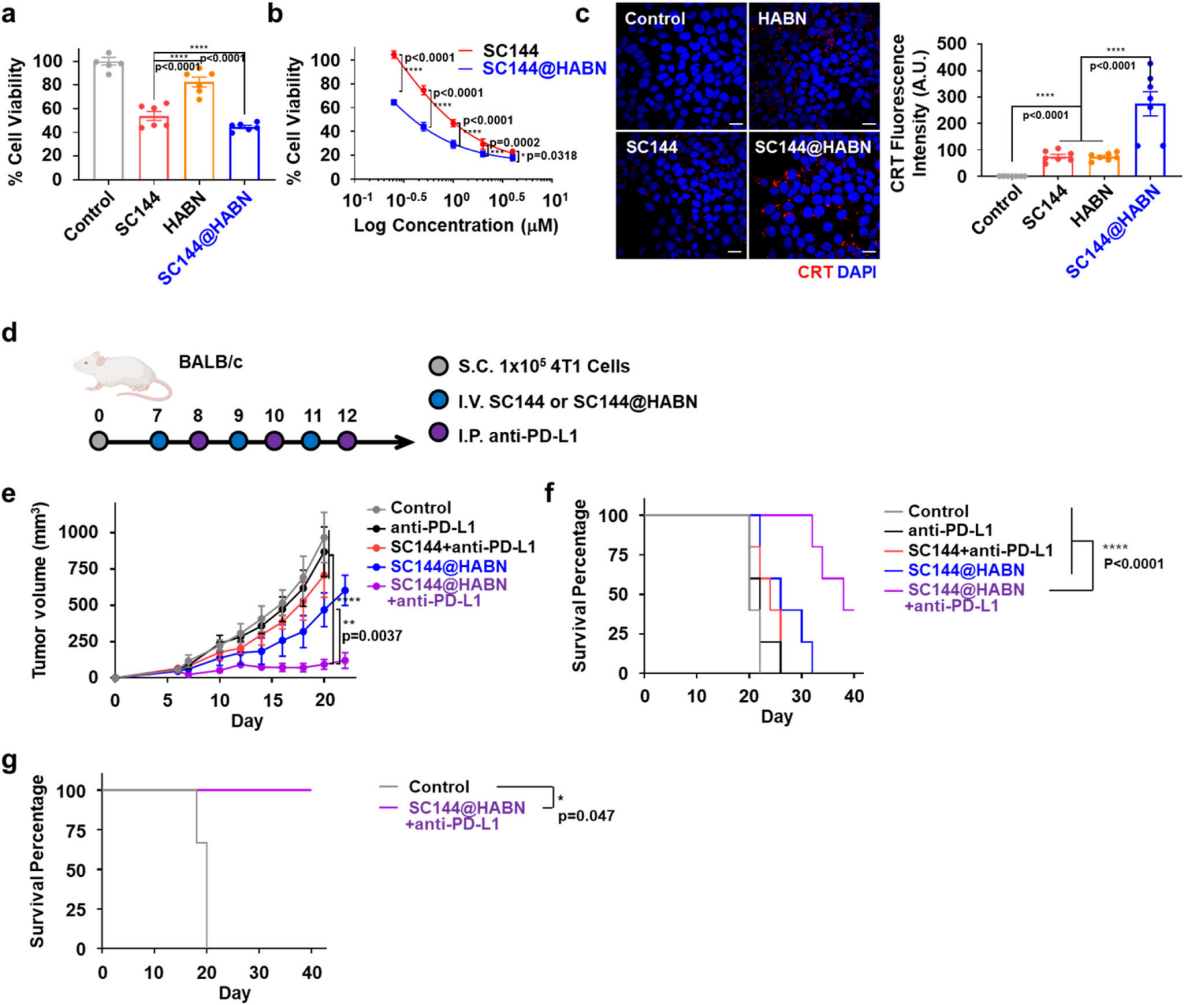
SC144@HABN and anti-PD-L1 combo therapy exerts robust anti-tumor efficacy in 4T1 tumor model. **a**, **b** Viability of 4T1 cells measured with CCK-8 assay after 24 h treatment with 10 μM of SC144, 40 μg/ml of HABN, SC144@HABN (10 μM of SC144; 40 μg/ml of HABN), or PBS. **c** Confocal microscopy images and CRT fluorescence intensities of MC38 cells pre-treated with SC144 (10 μM), HABN (40 μg/ml), SC144@HABN (10 μM of SC144; 40 μg/ml of HABN), or control medium for 20 h and stained with anti-CRT antibody. Scale bar = 20 μm. **d**–**f** BALB/c mice bearing 4T1 tumor were administered IV with SC144 (5 mg/kg), HABN (50 mg/kg), SC144@HABN (5 mg/kg of SC144; 50 mg/kg of HABN), or PBS on days 11, 13, and day 15 with or without intraperitoneal administration of anti-mouse PD-L1 (5 mg/kg) on days 12, 14, and 16. **d** 4T1 tumor growth curves and **f** survival of mice treated with the indicated formulations. **g** Tumor-free survivors were re-challenged with 4T1 tumor cells, and animal survival was monitored. The data represent mean ± s.e.m., biological replicates with *n* = 3 (**g**), 5 (**e**, **f**), 6 (**a**, **b**), or 7 (**c**). *p < 0.05, **p < 0.01, ***p < 0.001 analyzed by one-way ANOVA (**a**, **c**) or two-way ANOVA (**b**, **e**) with Tukey’s HSD multiple comparison post hoc test (**a**, **b**, **c**, **e**), or Kaplan–Meier survival analysis with the log-rank (Mantel–Cox) test (**f**, **g**). Source data are provided as a Source data file.

To validate our approach, we examined the anti-tumor efficacy of SC144@HABN + anti-PD-L1 ICB therapy in BALB/c mice bearing 4T1 tumor, which is an aggressive breast cancer model that is resistant to anti-PD-L1 and other ICB therapies^22^. First, we evaluated the cytotoxicity of SC144@HABN in 4T1 cells. Both SC144 and SC144@HABN induced killing of 4T1 cells in vitro (Fig. 5a, b). Notably, SC144@HABN induced upregulation of CRT on 4T1 cells, indicating SC144@HABN-mediated ICD (Fig. 5c) Moreover, 4T1 cells expressed higher level of CD44, compared with MC38 cells (Supplementary Fig. 22), suggesting that CD44-expressing 4T1 cells may be a good target for HABN-mediated delivery of SC144.

Next, we examined the anti-cancer activity of SC144@HABN + anti-PD-L1 combination therapy in 4T1 tumor model. BALB/c mice were inoculated at subcutaneous flank with 10^5^ 4T1 cell tumors on day 0, and when tumors were ~50 mm^3^ on day 7, animals were treated on days 7, 9, and 11 with IV administration of SC144@HABN, followed by anti-PD-L1 therapy (Fig. 5d). SC144@HABN + anti-PD-L1 combo therapy exerted potent anti-tumor efficacy, as shown by significantly reduced tumor growth, compared with SC144 + anti-PD-L1 or anti-PD-L1 monotherapy (*p* < 0.0001) as well as SC144@HABN monotherapy (*p* < 0.01) (Fig. 5e). Moreover, SC144@HABN + anti-PD-L1 also significantly improved animal survival with ~35% mice showing complete response, compared with all other control groups (*p* < 0.0001, Fig. 5f). In addition, all survivors in the SC144@HABN + anti-PD-L1 combo group were resistant to re-challenge with 4T1 tumor cells performed on day 50, demonstrating establishment of long-term anti-tumor immunity (Fig. 5g). Lastly, we showed that SC144@HABN + anti-PD-L1 combination did not trigger any overt signs of systemic toxicity, autoimmunity, or pathologies in the major organs (Supplementary Fig. 23).

Taken together, these results demonstrate that SC144@HABN + anti-PD-L1 antibody exerts potent anti-tumor efficacy with long-lasting anti-tumor immunity with minimal toxicity.

## Discussion

Here, we report our discovery that SC144 polarizes macrophages into M1-like phenotype (Supplementary Figs. 8, 10, 11) and induce ICD of cancer cells in vitro (Fig. 2f–i). Yet, SC144 also induced killing of CD8 + T-cells (Supplementary Fig. 16), which is a crucial cell type for anti-tumor immunity. Thus, in this work, we sought to deliver SC144 selectively to tumor cells and TAMCs, while sparing CD8 + T-cells from SC144-mediated cytotoxicity. Toward this goal, we have developed SC144@HABN. This was motivated by our observation that HABN administered IV accumulated in tumor cells and TAMCs in vivo (Fig. 1), potentially due to the interaction between HA coating of HABN and its ligand CD44 (Fig. 1i–l, Supplementary Figs. 5–7)^41^. Indeed, SC144@HABN administered IV was taken up by TAMCs, especially among CD44^hi^ TAMCs, resulting in conversion of immunosuppressive TAMCs into a less immunosuppressive phenotype, as shown by an increased ratio of M1-like to M2-like macrophages and reduced frequency of MDSCs in the TME (Fig. 2a–e, Supplementary Figs. 5, 6). In addition, SC144@HABN triggered apoptosis and ICD of tumor cells, while encapsulation of SC144 within HABN shielded CD8^+^ T-cells from SC144-mediated cytotoxicity (Fig. 2f–l, Supplementary Figs. 13–17), potentially due to the cytoprotective activity of HABN^38^. SC144@HABN treatment promoted strong tumor-infiltration of CD8^+^ T-cells and increased their proliferation and functionality in vivo, as shown by increased expression of Ki67^+^ and granzyme B^+^ on CD8^+^ T-cells (Fig. 3d–f).

The TME-modulating effects of SC144@HABN led to significantly improved anti-cancer activity, as compared against SC144 or HABN monotherapy (Fig. 3a–c). Nevertheless, SC144@HABN treatment was associated with increased expression levels of PD-1 among CD8^+^ T-cells and PD-L1 on tumor cells (Fig. 3g, I, j), suggesting exhaustion of tumor-infiltrating CD8^+^ T-cells. To counter such responses, we combined SC144@HABN with anti-PD-L1 therapy, leading to amplification of anti-tumor CD8^+^ T-cell responses with robust anti-tumor efficacy in both MC38 and 4T1 tumor models (Figs. 4, 5). In addition, tumor-free survivors in both MC38 and 4T1 tumor models were resistant to tumor re-challenge, indicating long-lasting adaptive immunity (Figs. 4l, m, 5g). Moreover, as 4T1 tumor model is highly resistant to ICB therapies^22^, our work highlights the potential of SC144@HABN to sensitize ICB-resistant tumors to ICB therapy.

In conclusion, our work shows that SC144@HABN converts TAMCs into a less immunosuppressive phenotype and triggers ICD of tumor cells, leading to robust anti-tumor effects and strong synergy with ICB therapy (Supplementary Fig. 24). While the exact molecular mechanism of action for SC144-mediated polarization of macrophages and ICD induction is beyond the scope of this work, prior literature suggests that the IL-6/gp130/STAT3 axis plays a crucial role in tumor progression and immunosuppression^29–34^. Thus, our work based on a gp130 inhibitor, SC144, may be applicable to a wide range of cancers that are resistant to ICB therapy. In addition, our work represents a new, generalizable nanoparticle-based strategy that can be applied to other chemotherapeutic agents with ICD-inducing or TME-modulating properties.

## Methods

### Synthesis of hyaluronic acid-bilirubin conjugate (HA-BR) and hyaluronic acid-cholesterol conjugate (HA-Chol)

Before synthesizing hyaluronic acid-bilirubin conjugate (HA-BR), an acid form of hyaluronic acid (HA) from hyaluronic acid sodium salt (Lifecore Biomedical) and an aminoethylene-bilirubin conjugate (AE-BR) were prepared. The acidic form of hyaluronic acid (HA) from hyaluronic acid sodium salt was prepared by dialysis against 0.01 M HCl for overnight, followed by lyophilization. To prepare AE-BR, 750 µmol of bilirubin (Lee Biosolutions) and 520 µmol of N-hydroxysuccinimide (NHS, Sigma-Aldrich) were added to 7.5 ml of dimethyl sulfoxide (DMSO) containing 0.225 µl of trimethylamine (TEA). Subsequently, 337.5 µmol of EDC [1-Ethyl-3-(3-dimethylaminopropyl)carbodiimide] (Sigma-Aldrich) was added to the mixture. After stirring for 10 min at room temperature (RT), 562.5 µmol of ethylenediamine (EDA) was added to the mixture, and the reaction was allowed to proceed with stirring for 4 h at RT under nitrogen gas. 50 ml of chloroform was added to the mixture and then washed twice with 50 ml of 0.1 M HCl, 0.1 M NaHCO_3_ and then water. After evaporating the chloroform solution, 45 ml of methanol was added to the reaction mixture and then centrifuged at 3000 × *g* for 10 min. The supernatant was then evaporated to yield AE-BR.

To synthesize hyaluronic acid-bilirubin conjugate (HA-BR) or hyaluronic acid-cholesterol conjugate (HA-Chol), 80 µmol of an acidic form of HA, 40 µmol of NHS were added to 4.8 ml of DMSO and then, after adding of 140 µmol of EDC, the mixture was stirred for 10 min at RT. Subsequently, 20 µmol of AE-BR, or 5 µmol of cholesterol-PEG-NH_2_ (Nanosoft Polymers) was added and stirred with the mixture for overnight at RT under nitrogen gas. The mixture was slowly poured into 30 ml of 0.01 M NaOH, and then dialysis was performed against 0.01 M NaOH for 5 h. Further dialysis was performed against 1:1 ratio of water/acetonitrile three times for 1 day, followed by distilled water three times for 2 days. The resulting solution was lyophilized, yielding HA-BR (native sodium salt form; 26.5 µg/ml of BR in 1 mg/ml of HABN) or HA-chol. ^1^H-NMR spectra were obtained on a Varian 500 MHz system (Varian); chemical shifts represent ppm downfield from tetramethylsilane. Bilirubin portion of HA-BR or HA-Chol was calculated by measurement of UV/VIS spectra using Synergy^TM^ NEO HTS multi-mode microplate reader (BioTek Instruments Inc).

### Synthesis of PEGylated bilirubin (PEG-BR)

75 µmol of bilirubin and 33.75 µmol of EDC were added to 0.6 ml of DMSO containing 225 µl of TEA and 52 µmol of NHS for 10 min at RT. After then, 15 µmol of polyethylene glycol 20K-amine (PEG-NH_2,_ Nanocs) was added and mixed for 4 h under nitrogen gas. Subsequently, the mixture was added to 50 ml of chloroform, and the organic solvent was washed with 50 ml of 0.1 M HCl twice, followed by washing with 50 ml of 0.1 M NaHCO_3_ twice and washing with 50 ml of distilled water twice. The organic layer was evaporated, and 50 ml of methanol was added to the residue. After centrifugation at 3000 × *g* for 10 min, the supernatant was collected and then evaporated. Dialysis was performed as described in the previous section. To yield PEGylated bilirubin (PEG-BR), lyophilization was performed. The final structure was confirmed by ^1^H-NMR. ^1^H-NMR spectra were recorded on a Varian 500 MHz system; chemical shifts represent ppm downfield from tetramethylsilane.

### Synthesis of HA-BR-Cy5.5 conjugate

Prior to conjugating HA-BR with Cy5.5 amine (AAT Bioquest), a native sodium salt form of HA-BR was converted into an acidic form of HA-BR, HA-Chol or free HA by dialysis processes for efficient dissolution in DMSO. 10 µmol of each power form was dissolved into 5 ml of distilled water. Dialysis was performed against 0.1 M HCl for overnight, and then the solution was dialyzed against distilled water three times for 1 day. The resulting powder was obtained through a lyophilization step. 10.5 µmol of the acidic form of HA-BR, HA-Chol, or free HA was dissolved in 0.8 ml of DMSO for overnight. 2 µmol of NHS and 2 µmol of EDC were added to the mixture. After mixing for 10 min at RT, 0.1 µmol of Cy5.5-NH_2_ was further added to the reaction mixture. After stirring for overnight at RT, dialysis was performed once more against 0.01 M NaOH three times for 1 day, followed by distilled water twice for 2 days. After lyophilization, the native sodium salt form of HA-BR-Cy5.5 conjugate was obtained.

### Preparation of hyaluronic acid-bilirubin nanoparticles (HABN), hyaluronic acid-cholesterol nanoparticles (HACN), PEGylated bilirubin nanoparticles (PEG-BN), or Cy5.5-tagged HABN

After dissolving HA-BR, HA-Chol, PEG-BR, or HA-BR-Cy5.5 in water or PBS, ultra-sonication (140 W, 26 Hz, 2 s on and 3 s off of short interval) was performed for 5 min at 4 °C. After filtration through a 0.45 µm of filter membrane, HABN, HACN, PEG-BN or Cy5.5 conjugated HABN (HABN-Cy5.5) were acquired. The size and zeta potential of the nanoparticles were obtained in Zetasizer software (v 7.13, Malvern Instruments Ltd) using a Nanosizer ZS90 Malvern Instruments Ltd). Morphology was examined by Transmission Electron Microscopy (TEM) using JEOL 1400-plus TEM (JEOL USA). The resulting nanoparticles were diluted in PBS or culture medium for in vitro and in vivo experiments.

### Preparation of SC144-loaded HABN (SC144@HABN)

SC144-loaded HABN (SC144@HABN) was prepared using an o/w emulsion method. Briefly, 15 mg of HABN was dissolved in 1.5 mL of sterilized distilled water and stirred for 5 min at room temperature. After adding 60 μl of SC144 (5 mg/100 μl DMSO) dropwise to the solution, ultra-sonication (140 W, 26 Hz, 2 s on and 3 s off of short interval) was performed for 5 min at 4 °C. The solution was dialyzed against an excess amount of distilled water and acetonitrile with a dialysis bag overnight, followed by centrifugation at 14,000 rpm for 10 min to concentrate. Before further analysis, the solution was filtered through a 0.45 μm pore-sized microporous membrane. The size and zeta potential of the nanoparticles were obtained using a Nanosizer ZS90 (Malvern Instruments Ltd). Morphology was examined by Transmission Electron Microscopy (TEM) using JEOL 1400-plus TEM (JEOL USA), and TEM images were acquired with AMT602 software (JEOL USA). The resulting nanoparticles were diluted in PBS or culture medium for in vitro and in vivo experiments. SC144-loaded HACN and SC144-loaded HACN were also prepared using the same o.w emulsion method as described above. The amount of SC144 in each nanoparticle formulation was determined by using HPLC and calculated as shown below.

Encapsulation efficiency (%) = (weight of drug in particle/weight of drug added initially) × 100.

Drug loading percentage (%) = [weight of drug in particle/(weight of drug in particle + weight of HABN added initially)] × 100.

### Animals

Animals were cared for following federal, state, and local guidelines. All work performed on animals was in accordance with and approved by the Institutional Animal Care & Use Committee (IACUC) at University of Michigan, Ann Arbor and Ewha Womans University (EWHA IACUC 21-068-4). All animals were obtained from the Jackson Laboratory (Bar Harbor, ME) and the Laonbio (South Korea) as mixed littermates. All animals were housed under pathogen-free conditions with controlled temperature (20–26 °C), humidity (40–60%), and lighting (12-h light-dark cycle) in the animal facility at the North Campus Research Complex of University of Michigan and the College of Pharmacy, Ewha Womans University, respectively. Mouse tumor size and survival were monitored every 2–4 days. Tumor size was calculated based on the equation: volume = length × width^2^ × 0.5. Animals were euthanized when the tumor reached 2.0 cm in diameter or when they became moribund with more than 20% weight loss or unhealing ulceration. During all the animal studies, the maximum tumor size permitted by the IACUC at University of Michigan, Ann Arbor and Ewha Womans University was not exceeded.

### In vivo IVIS imaging

To check ability of HABN to target the tumor tissue, C57BL/6 mice were inoculated subcutaneously with 3 × 10^5^ MC38 cells on day 0. After treating animals of with 10 mg/kg of HABN-Cy5.5 (having the equivalent mass of free Cy5.5) or 0.25 mg/kg of free Cy5.5 on day 25, whole body fluorescence intensities were monitored at predetermined times using a Xenogen IVIS Lumina in vivo imaging system (PerkinElmer) with a Cy 5.5 filter channel and an exposure time of 5 s. In vivo images were acquired and analyzed using IVIS Lumina Living Image Software (v.4.5.5, PerkinElmer). After 24 h of treating animals of each group, mice were euthanized, and organs including heart, lung, kidney, spleen, liver, and tumor were excised. Fluorescence intensity in organs from each group was analyzed using a Xenogen IVIS Lumina in vivo imaging system (PerkinElmer) with a Cy 5.5 filter channel and an exposure time of 5 s. For the HABN-Cy5.5 uptake studies in vivo, animals were euthanized on day 12, and tumor tissues harvested, followed by flow cytometric analysis.

### In vivo tumor study

For studies with MC38 colon carcinoma, C57BL/6 mice were inoculated subcutaneously with 3 × 10^5^ MC38 cells or 3 × 10^5^ MC38 CD44 knock-out cells on day 0 and injected IV with SC144 (5 mg/kg), HABN (50 mg/kg), SC144@PEG-BN (5 mg/kg of SC144; 50 mg/kg of PEG-BN), SC144@HACN (5 mg/kg of SC144; 50 mg/kg of PEG-BN), SC144@HABN (5 mg/kg of SC144; 50 mg/kg of HABN), or PBS on days 11, 13, and 15 with or without intraperitoneal administration of anti-CSF1R (AFS98, Bioxcell, #BE0213, 20 mg/kg) on days 7, 9, 11, 13, and 15; anti-mouse IL-6 (MP5-20F3, Bioxcell, #BE0046, 10 mg/kg) on days 12, 14, and 16; or anti-mouse PD-L1, (10F.9G2, Bioxcell, #BE0101, 5 mg/kg) on days 12, 14, and 16. Tumor growth was monitored every other day, and the tumor volume was calculated by the following equation: tumor volume = (length × width^2^)/2. When individual tumor mass reached 15 mm in diameter in any dimension or when animals became moribund with severe weight loss or active ulceration, animals were euthanized. For the tumor microenvironment study, animals were euthanized, and tumor tissues harvested on day 18. For acquiring in vivo confocal microscopy images, animals were euthanized on day 22, and 5 cm of tumor tissue was placed in OCT cryomold blocks and transferred to −70 °C freezer. Terminal deoxynucleotidyl transferase (TdT) dUTP Nick-End Labeling (TUNEL) assay was performed to detect apoptosis in OCT-embedded frozen blocks according to the manufacturer’s instructions. For treatment studies involving animals injected with 4T1 cells, BALB/c mice were inoculated with 1 × 10^5^ 4T1 cells in the mammary fat pad on day 0, and injected IV with SC144 (5 mg/kg), SC144@HABN (5 mg/kg of SC144; 50 mg/kg of HABN), or PBS on days 12, 14, and day 15 with or without intraperitoneal administration of anti-mouse PD-L1 (5 mg/kg) on days 8, 10, and 12. For tumor re-challenge study, mice that cleared primary MC38 or 4T1 tumors were re-challenged by subcutaneous injection of 3 × 10^5^ of MC38 or mammary fat pad injection of 1 × 10^5^ 4T1 cells on day 60, and subsequent tumor growth was monitored as described above. Naïve mice were used as controls.

### Flow cytometric analysis

For a subset of studies, tumor tissues harvested on the indicated time points were cut into small pieces of 2 to 4 mm, and cells were dissociated in digestion buffer [collagenase type IV (1 mg/ml) and deoxyribonuclease I (100 U/ml) in serum-free RPMI] for 30 min at 37 °C with gentle shaking. This cell suspension was passed through a 70-μm nylon strainer and washed with FACS buffer. Cells were pre-incubated with a FcγR-blocking mAb (1/20 dilution, #14-0161-82, CD16/32; 93, eBioscience) for 10 min, followed by incubation with specific mAbs for 30 min on ice. After staining surface molecules, the cells were re-suspended in fixation/permeabilization solution (eBioscience), and intracellular staining of FOXP3 was performed with a FOXP3 staining buffer kit (eBioscience). Flow cytometric analyses were performed on CytoFLEX Cell Analyzer with CytExpert Software (v 2.2.0.97) (Beckman Coulter), and data were analyzed using Flowjo software (v.10.5, Tree Star). Background fluorescence was assessed by staining with isotype-matched control mAbs. FITC-, PE-, BV605-, PerCP-Cy5.5-, APC-, PE-Cy7-, or APC-Cy7- conjugated mAbs against, CD3 (17A2), CD11b (M1/70), CD11c (N418), Ly6c (HK1.4), Ly6G (1A8), MHCII (M5/114.15.2), CD45 (30-f11), CD206(C068C2), F4/80 (BM8), PD-1 (RMP1-30), PD-L1 (10F.9G2), and Ki67 (16A8). Antibodies (1:100 dilution) were purchased from Biolegend. CD4 (1:100 dilution, RM4-5, eBioscience), Granzyme B (1:100 dilution, NGZB, ebioscience), CD44 (1:100 dilution, IM7, eBioscience), FOXP3 (1:100 dilution, FJK-16S, eBioscience), eflour 450 (eBioscience) were purchased from eBioscience. CD8 (1:100 dilution, 53-6.7, BD bioscience) and CRT (1:100 dilution, FMC 75, abcam) were purchased from BD bioscience and Abcam, respectively. For HABN-Cy5.5 in vivo uptake studies the following antibodies, all purchased from Biolegend, were used: CD45-BV421 (1:200 dilution, #103133, 30-F11), MHC-II-Pacific Blue (1:200 dilution, #107619, M5/114.15.2), CD44-BV510 (1:200 dilution, #103039, IM7), Ly6C-BV711 (1:200 dilution, #128037, HK1.4), CD11b-FITC (1:200 dilution, #101205, M1/70), CD206-PE (1:200 dilution, #141705, C068C2), and F4/80-PE-Cy7 (1:200 dilution, #123113, BM8). HABN-Cy5.5 uptake tumor samples were run on a Cytek Aurora with SpectroFlo software (v 3.0), and data were analyzed in Flowjo (v.10.5 Tree Star). Catalog numbers of all the antibodies were presented in the Supplementary Table 1 and reporting summary.

### In vivo ELISA analysis

For determining the concentrations of cytokines in the serum, blood was collected at indicated time points and allowed to clot for 30 min, and the serum was collected by centrifugation for 10 min at 3000 × *g* in the serum separator microtainer (BD Science). CXCL-10 concentration in the serum was measured by enzyme-linked immunosorbent assay (ELISA) at the Cancer Center Immunology Core of the University of Michigan. HMGB1 was measured using a mouse HMGB1 ELISA kit (LifeSpan BioSciences Inc.).

### Cell culture

Bone marrow-derived macrophages, BMDMs, were prepared described previously^43^. Briefly, cells were aseptically isolated from femurs and tibia of C57BL/6 mice and cultured in complete DC media comprised of RPMI-1640 (Gibco) supplemented with 10% fetal bovine serum (FBS, Corning), 1% penicillin/streptomycin (Gibco), 55 μM β-mercaptoethanol (Gibco), and 20 ng/mL granulocyte–macrophage colony-stimulating factor (GM-CSF, Genscript) at 37 °C with 5% CO_2_. Fresh media was added on day 3, and all media was renewed on days 6 and 8. The cultured BMDCs were used between days 7 and 12. CT26, MC38, and 4T1 cell lines were obtained from the American Type Culture Collection (ATCC). CD44-KO MC38 cells were prepared using a mixture of CRISPR/Cas9 mRNA (1 μg/ml) and CD44 gRNA (Axolabs, 1 μg/ml) complexed with lipofectamine MessengerMAX (3:1000 ratio, Thermofisher). All cell lines have been tested negative for mycoplasma contamination. BMDM, CT26, MC38, or 4T1 cells in RPMI medium (Gibco^TM^) containing 10% (v/v) heat-inactivated fetal bovine serum (FBS), 100 IU/ml penicillin/streptomycin, and L-glutamine were cultured in a humidified 5% CO_2_ atmosphere at 37 °C.

### In vitro confocal microscopy

BMDM, CT26, MC38, or 4T1 cells in culture medium were seeded onto cover slip in 24-well plates (1.0 × 10^5^/well). For the uptake study with BMDM, after incubation for 2 days at 37 °C, cells were treated for 24 h with 100 ng/ml of LPS and 10 ng/ml of IFN-γ for M1 induction, 20 ng/ml of IL-4 for M2 induction, or control medium. For CRT or PD-L1 marker study, MC38 or 4T1 cells in culture medium were treated with SC144 (10 μM), HABN (40 μg/ml) or SC144@HABN (10 μM of SC144; 40 μg/ml of HABN) for 20 h. For preparation of confocal samples before confocal microscopy analysis, 20 µg/ml of HABN-Cy5.5, FITC conjugated anti-CD44 antibody (1:100 dilution), PE-conjugated calreticulin antibody (1:100 dilution), PE-conjugated anti-PD-L1 antibody (1:100 dilution) or control medium was treated with the cells for 1 h. The cells were then fixed with 4% PFA for 5 min, counter-stained with Hoechst 33342 for 10 min and analyzed by confocal laser-scanning microscopy (Nikon A1). Confocal fluorescence images were acquired and analyzed by Nikon A1 NIS elements Imaging software (v.5.02). For the particle uptake competition assay, 2 mg/ml of free HA was pre-treated with the cells for 30 min before nanoparticle treatment. For the CD44 siRNA assay, a mixture of anti-CD44 siRNA (10 nM, stealth RNAi siRNA, Thermofisher, #4390771) and lipofectamine 2,000 (1:100 Opti-MEM reduced serum medium, Thermofisher) was pre-treated with the cells for 2 days in Opti-MEM reduced serum medium followed by 20 ng/ml of IL-4 treatment or control-medium overnight.

### In vitro ELISA analysis

For determining the concentrations of cytokines (IL-1β, IL-6, TNF-α, IL-10, TGF-β), BMDM cells were treated with SC144 (10 μM), HABN (40 μg/ml), or SC144@HABN (10 μM of SC144; 40 μg/ml of HABN), or fresh medium for 24 h in the absence or presence of 20 ng/ml of IL-4. As M0 and M1 control groups, the cells were treated with 100 ng/ml of LPS and 10 ng/ml of IFN-γ for M1 induction, or control medium for M0 control. The levels of cytokines in the resulting supernatants or medium were measured by ELISA at the Cancer Center Immunology Core of the University of Michigan. FACS analysis was also performed to check macrophage phenotypes. To measure the release of CXCL10 and HMGB1 from dying tumor cells, MC38 cells seeded in 96-well plates (5 × 10^3^/well). After 1 day incubation, cells were incubated with SC144 (10 μM), HABN (40 μg/ml) or SC144@HABN (10 μM of SC144; 40 μg/ml of HABN) for 24 h. After incubation, each supernatant was collected and centrifuged at 1000 × *g* for 20 min. HMGB1 was measured using a mouse HMGB1 ELISA kit (LifeSpan BioSciences Inc.).

### CCK-8 assay

BMDM, CT26, MC38, or 4T1 cells in culture medium were grown in 96-well plates (0.7 × 10^4^/well) for 24 h at 37 °C. After medium was removed, SC144 (10 μM), HABN (40 μg/ml), SC144@HABN (10 μM of SC144; 40 μg/ml of HABN), or fresh medium was added to each well and plates were incubated for 24 h. After incubation, cells were washed with fresh culture medium and 100 µl of fresh culture medium was added to each well, followed by the addition of 10 µl of CCK-8 (Dojindo Molecular Technologies. Inc.) and after incubation for 1 h at 37 °C, the absorbance was measured at 450 nm using a 96-well plate microreader.

### In vitro FACS analysis

BMDM, CT26, MC38, or 4T1 cells in culture medium were grown in 6-well plates (1 × 10^6^/well) for 24 h at 37 °C. After medium was removed, SC144 (10 μM), HABN (40 μg/ml), SC144@HABN (10 μM of SC144; 40 μg/ml of HABN), or fresh medium was added to each well and plates were incubated for 24 h. Cells were pre-incubated with a FcγR-blocking mAb (1:20 dilution, CD16/32; 93, eBioscience) for 10 min, followed by incubation with specific mAbs for 30 min on ice. Flow cytometric analyses were performed on CytoFLEX Cell Analyzer with CytExpert Software (v 2.2.0.97) (Beckman Coulter), and data were analyzed using Flowjo software (v.10.5, Tree Star). Background fluorescence was assessed by staining with isotype-matched control mAbs. PE-conjugated PD-L1 (1:100 dilution, #124307, 10 F.9G2, Biolegend), FITC-conjugated CD44 (1:100 dilution, #11-0441-82, IM7, eBioscience), or PE-conjugated CRT (1:100 dilution, ab83220, FMC 75, abcam) were used. Annexin-V-FITC/PI staining was used to differentiate between early apoptotic cells, late apoptotic cells, necrotic cells, and viable cells.

Spleens from naïve mice were isolated and ground through 40-μm filters to generate a single cell suspension. After RBC lysis, CD8+ cells were purified using anti-CD8 (Ly-2) microbeads (Miltenyi Biotech) according to manufacturer’s protocol. CD8 + T cells were then plated in complete RPMI media supplemented with 0.05 M β-mercaptoethanol onto 24-well plates (0.3 × 10^5^ cells/well) coated with 1 μg/ml anti-CD3 (clone 1454-2C11) and 5 μg/ml anti-CD28 (clone 37 N) antibodies. SC144 (2 μM), HABN (8 μg/ml), or SC144@HABN (2 μM of SC144; 8 μg/ml of HABN) were added to the cells and plates were incubated at 37 °C for 1 h. After 24, 48, or 72 h, the live cell number in each sample was determined by flow cytometry (CytoFLEX Cell Analyzer, Beckman Coulter). The fold induction was then calculated by the fold ratio of live cells to the live cells on day 0.

For assessing the uptake of HABN-Cy5.5 or Free Cy5.5 among T cells, CD8^+^ T cells were isolated from spleens of naïve C57BL6 mice using StemCell’s CD8 Negative Selection Kit. Isolated T cells were activated with plate bound αCD3 (1 μg/mL) plus soluble αCD28 (0.5 μg/mL) and IL-2 (10 ng/mL) for 3 days. On the day of co-culture, spleens from naïve C57BL6 were collected, and CD8 T cells were isolated for use as not-activated CD8 + T cell controls. Then, 2 × 10^5^ freshly isolated, αCD3/CD28-activated, and not-activated total splenocytes were co-cultured with PBS, HABN-Cy5.5 (20 μg/mL), or Free-Cy5.5 (0.8 μg/mL) for 1 h. Uptake among CD44^hi^ vs. CD44^lo^ populations was quantified by flow cytometric analysis on a Cytek Aurora. The following antibodies were used: CD8-Pacific Blue (1:200 dilution, #100728, Clone: 53-6.7, Biolegend) and CD44-PE-Dazzle (1:200 dilution, #103055, Clone: IM7, Biolegend).

### In vivo toxicity test

Six-weeks old female C57-BL/6 mice were housed in groups of five mice per cage and acclimatized for 1 wk before inclusion in the study. 30 mg/kg of HABN (10 kDa, 100 kDa, or 700 kDa) or PBS was administered via an oral route on day 0, 2, 4, and 6. Mice were observed over a 1 wk period for changes in behavior or weight. Blood was collected from jugular vein and immediately sent to the ULAM Pathology Core for Animal Research for blind blood assessment. Mice were sacrificed, and their major organs (heart, liver, lung, kidney, spleen, and colon) were collected for histopathological analysis. Each organ was fixed with 4% (v/v) buffered formalin and 70% (v/v) alcohol, and embedded in paraffin. Tissues were sectioned, stained with H&E, and examined by microscopy. Histology images were acquired using Matra Quantitative Pathology Workstation (v.1.0.3) and analyzed by inForm image analysis software (v.2.3.0). All histological assessments were performed in a blinded manner to prevent observer bias.

### Statistical analysis

The results are expressed as means ± s.e.m. A one-way or two-way ANOVA, followed by Tukey’s HSD multiple comparison post hoc test was used for testing differences among groups. Data were approximately normally distributed and variance was similar between the groups. No samples were excluded from analysis. Statistical significance is indicated as *p < 0.05, **p < 0.01, ***p < 0.001, and ****p < 0.0001. GraphPad Prism 8.0 (GraphPad Software, La Jolla, CA) was used for statistical analyses. All experiments were repeated at least twice with similar results.

### Reporting summary

Further information on research design is available in the Nature Portfolio Reporting Summary linked to this article.

### Supplementary information

Supplementary Information

Reporting Summary

### Source data

Source Data

## Data Availability

The authors declare that data supporting the findings of this study are available within the article and its Supplementary Information files. Source data are provided with this paper.
