# Supplementary material for: Hyaluronic acid-bilirubin nanomedicine-based combination chemoimmunotherapy
Source: Nat Commun. 2023 Aug 8;14:4771. doi: 10.1038/s41467-023-40270-5 (PMC10409794; doi:10.1038/s41467-023-40270-5)
Supplement: Supplementary file 1 — Supplementary Information [file 41467_2023_40270_MOESM1_ESM.pdf]

Supplementary Information for

Hyaluronic acid-bilirubin nanomedicine-based combination chemoimmunotherapy.

Yonghyun Lee[1,2,3,4,*], Jongyoon Shinn[1,2], Cheng Xu[3,4], Hannah E. Dobson[3,4], Nouri Neamati[5], and James J. Moon[3,4,6,7,*]

[1]Department of Pharmacy, College of Pharmacy, Ewha Womans University, Seoul 03760, South Korea

[2]Graduate School of Pharmaceutical Sciences, Ewha Womans University, Seoul 03760, South Korea

[3]Department of Pharmaceutical Sciences, University of Michigan, Ann Arbor, Michigan 48109, USA.

[4]Biointerfaces Institute, University of Michigan, Ann Arbor, Michigan 48109, USA.

[5]Department of Medicinal Chemistry, University of Michigan, Ann Arbor, Michigan 48109 USA.

[6]Department of Biomedical Engineering, University of Michigan, Ann Arbor, Michigan 48109 USA.

[7]Department of Chemical Engineering, University of Michigan, Ann Arbor, Michigan 48109 USA.

*E-mail: y.lee@ewha.ac.kr and moonjj@umich.edu

Supplementary Figures

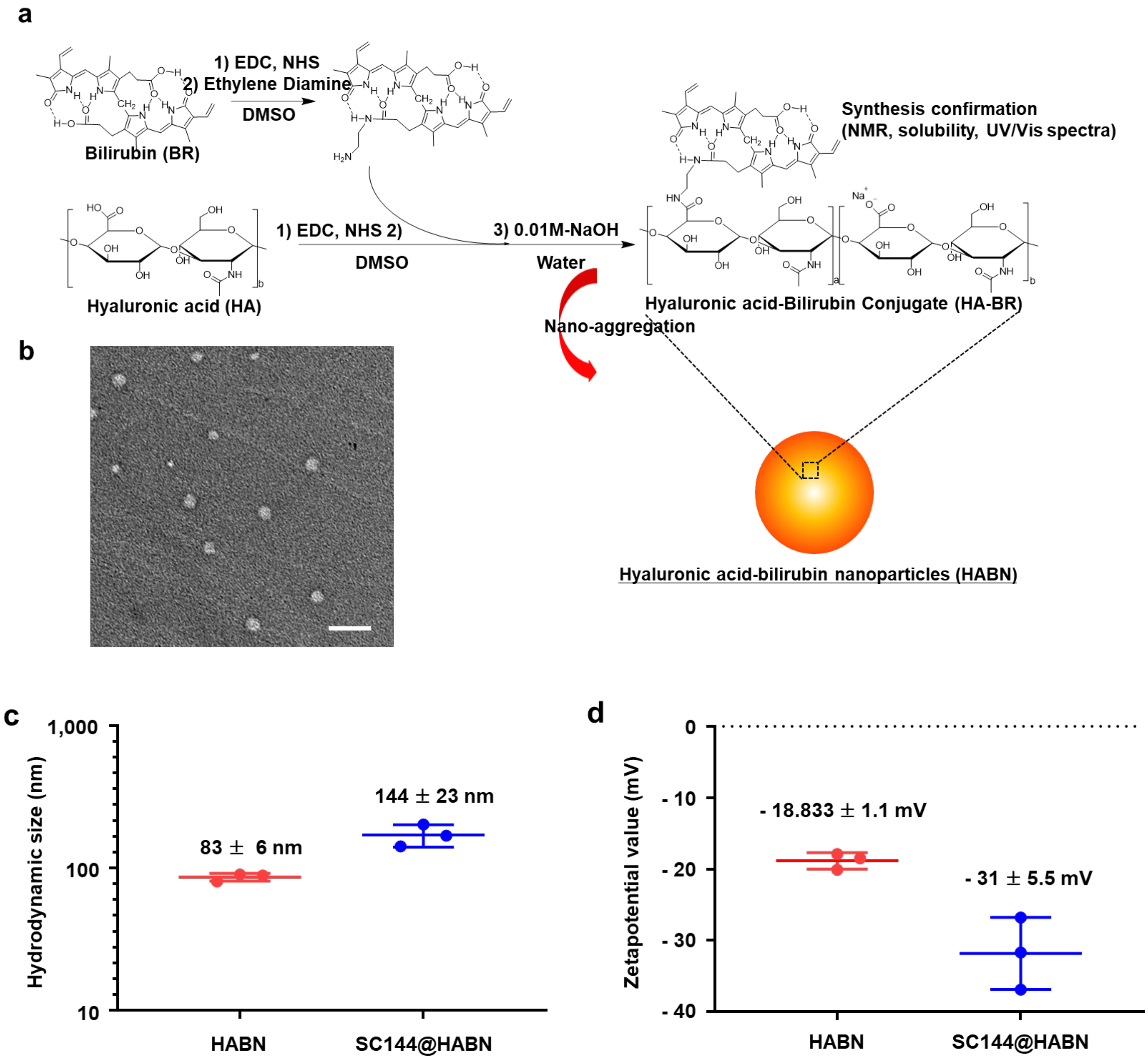

Supplementary Figure 1. Schematic illustration of HABN synthesis. a-c, Synthesis of hyaluronic acid-bilirubin conjugate (HA-BR, **a)** and hyaluronic acid-bilirubin nanoparticles (HABN) self-assembled from HA-BR and its TEM images **(b),** hydrodynamic sizes **(c),** and zeta-potential **(d).** Scale bar = 200 nm. The data represent mean ± s.e.m. with n = 3 batches.

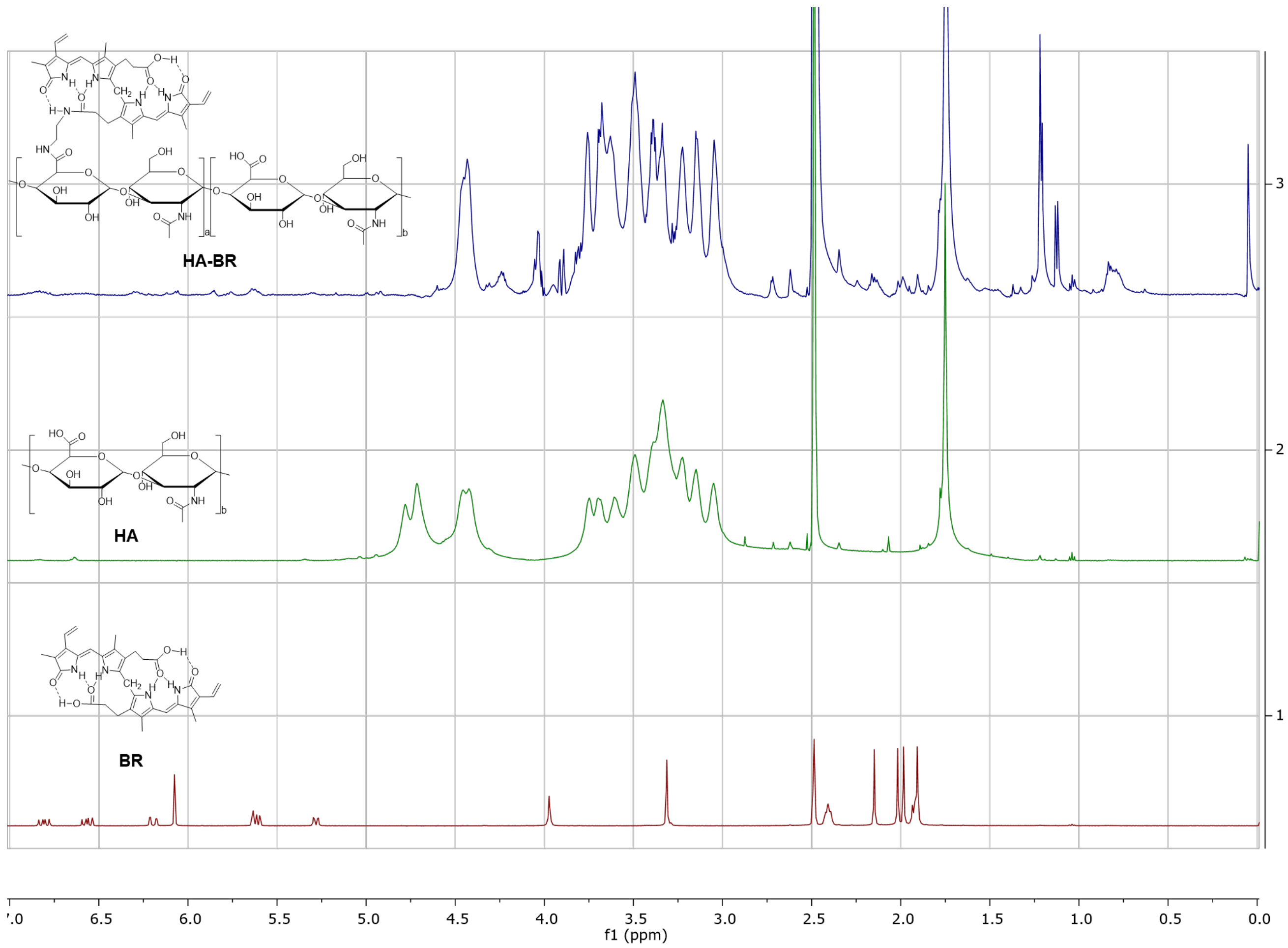

Supplementary Figure 2. **NMR spectra of HA-BR, hyaluronic acid (HA), and bilirubin (BR).**

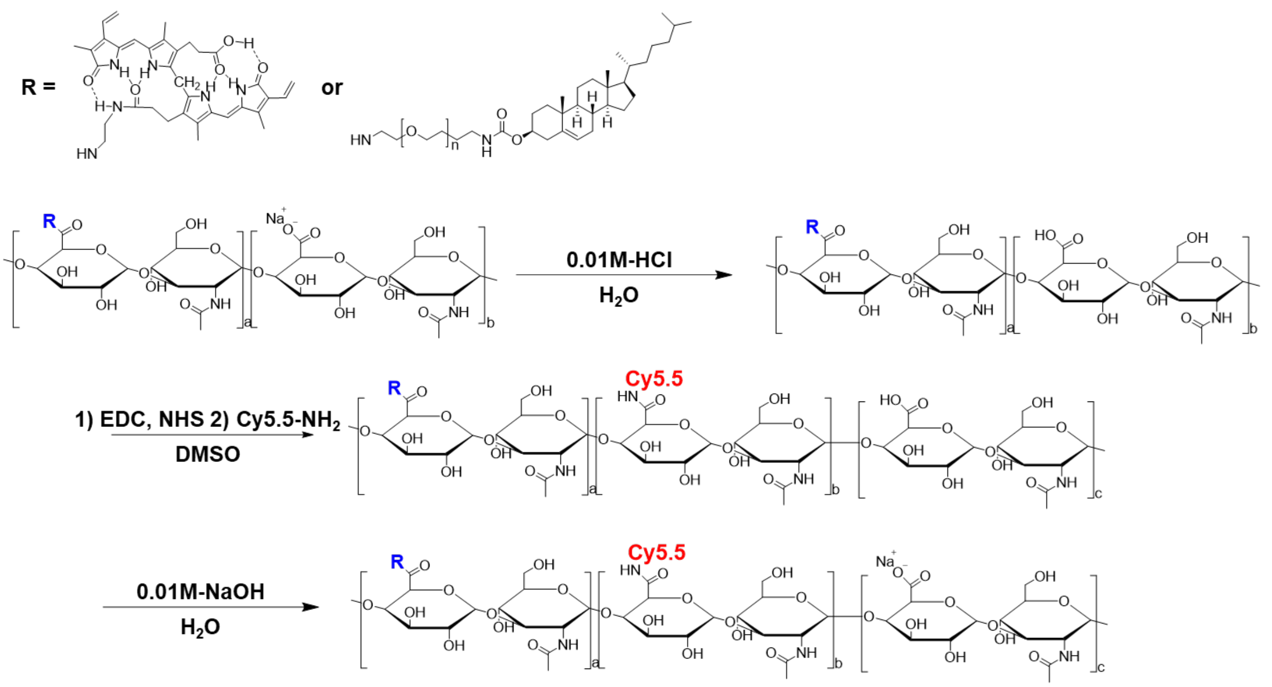

Supplementary Figure 3. **Schematic illustration of the synthesis of HABN-Cy5.5 and HACN-Cy5.5.**

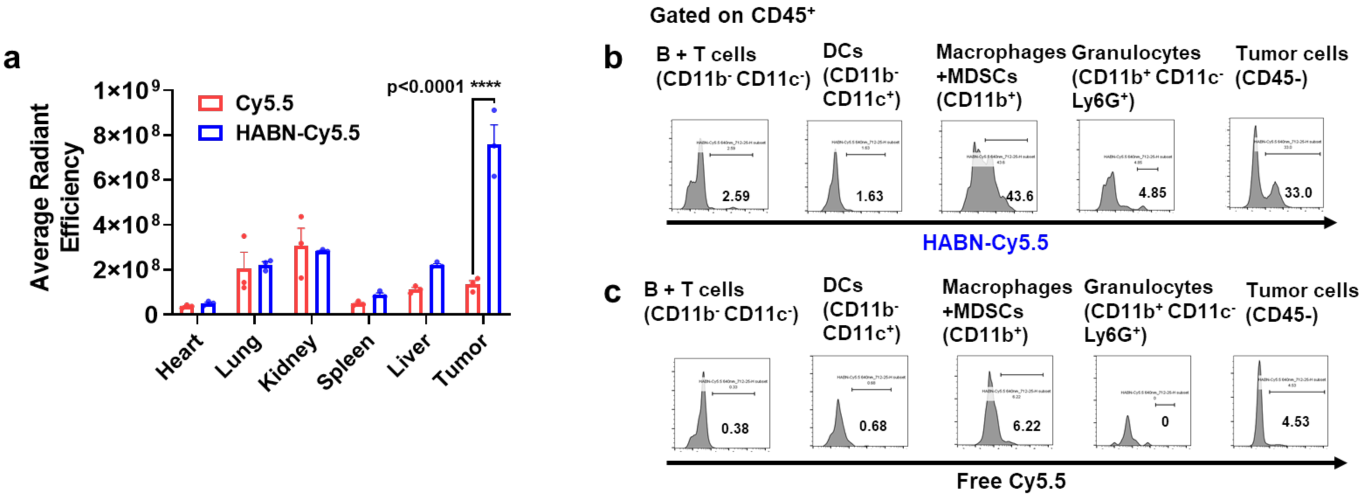

Supplementary Figure 4. **HABN accumulates in tumor cells and tumor-associated myeloid cells *in vivo*. a,** MC38 tumor-bearing mice were administered IV on day 25 with 10 mg/kg of HABN-Cy5.5, or 0.25 mg/kg of free Cy5.5 (equivalent mass of Cy5.5 in HABN-Cy5), followed by quantification of Cy5.5 fluorescence signal in various tissues by IVIS imaging. **b-c,** MC38 tumor-bearing mice were intravenously administered on day 25 with 10 mg/kg of HABN-Cy5.5 or 0.25 mg/kg of free Cy5.5 (equivalent mass of Cy5.5 in HABN-Cy5), followed by IVIS analysis and flow cytometry. Comparison of uptake levels of HABN-Cy5.5 (**a**) and free Cy5.5 (**b**) among various immune cells and cancer cells in tumor tissues. The data represent mean ± s.e.m. biological replicates with n = 3. ****p < 0.0001 analyzed by one-way ANOVA (**a**) with Tukey's HSD multiple comparison post hoc test.

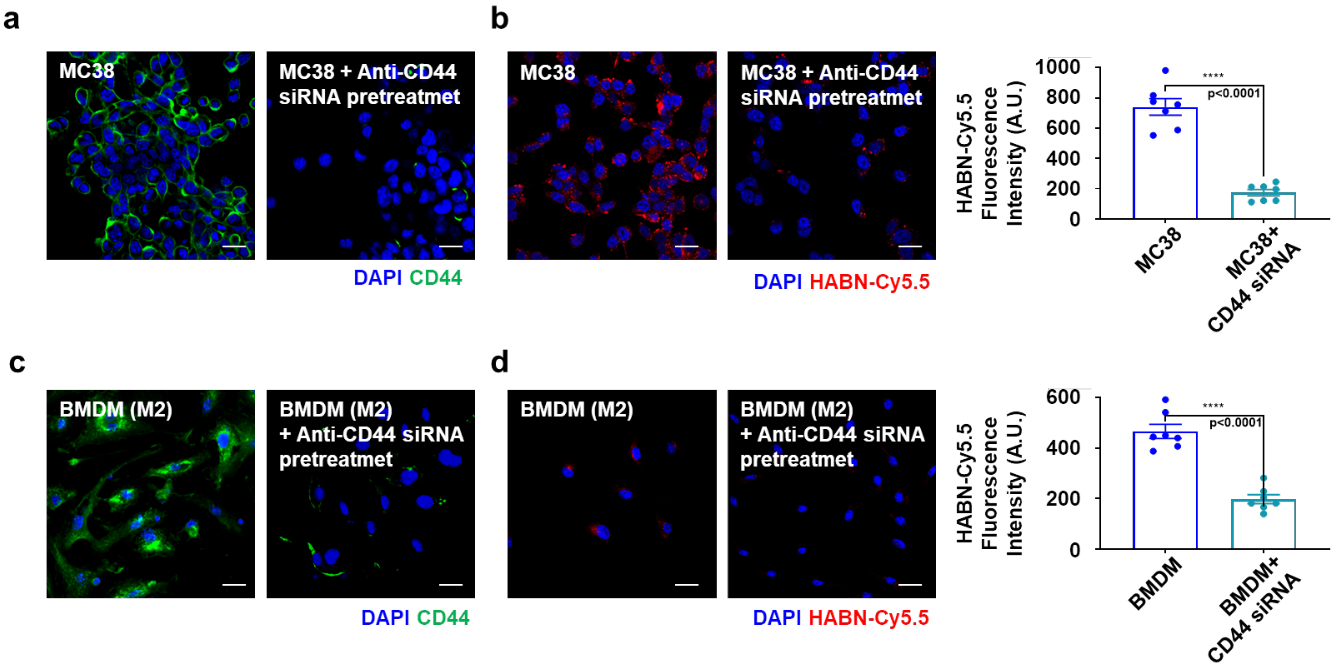

Supplementary Figure 5. **HABN is taken up by MC38 and BMDM cells in a CD44-dependent manner.** **a,** Confocal microscopy images of MC38 cells stained with anti-CD44 antibody. For the CD44 silencing, MC38 cells were pre-treated for 2 days with a mixture of lipofectamine 2,000 and anti-CD44 siRNA. **b,** Confocal microscopy images of MC38 cells incubated with HABN-Cy5.5 (20 μg/ml) for 1h and quantification of HABN-Cy5.5 fluorescence intensity. For the CD44 silencing, MC38 cells were pre-treated for 2 days with a mixture of lipofectamine 2,000 and anti-CD44 siRNA. **c,** Confocal microscopy images of BMDM cells pre-incubated with IL-4 (20 ng/ml) for 20 h and stained with anti-CD44 antibody. For the CD44 silencing, BMDM 38 cells were pre-treated for 2 days with a mixture of lipofectamine 2,000 and anti-CD44 siRNA. **d,** Confocal microscopy images and HABN-Cy5.5 fluorescence intensity in BMDM cells pre-incubated with IL-4 (20 ng/ml) for 20 h, followed by 1 hr treatment with HABN-Cy5.5 (20 μg/ml). For the CD44 silencing, BMDM cells were pre-treated for 2 days with a mixture of lipofectamine 2,000 and anti-CD44 siRNA before IL-4 treatment. Scale bars = 20 μm. The data represent mean ± s.e.m. biological replicates with n = 7. ****p < 0.0001, analyzed by two-sided Student's T-test.

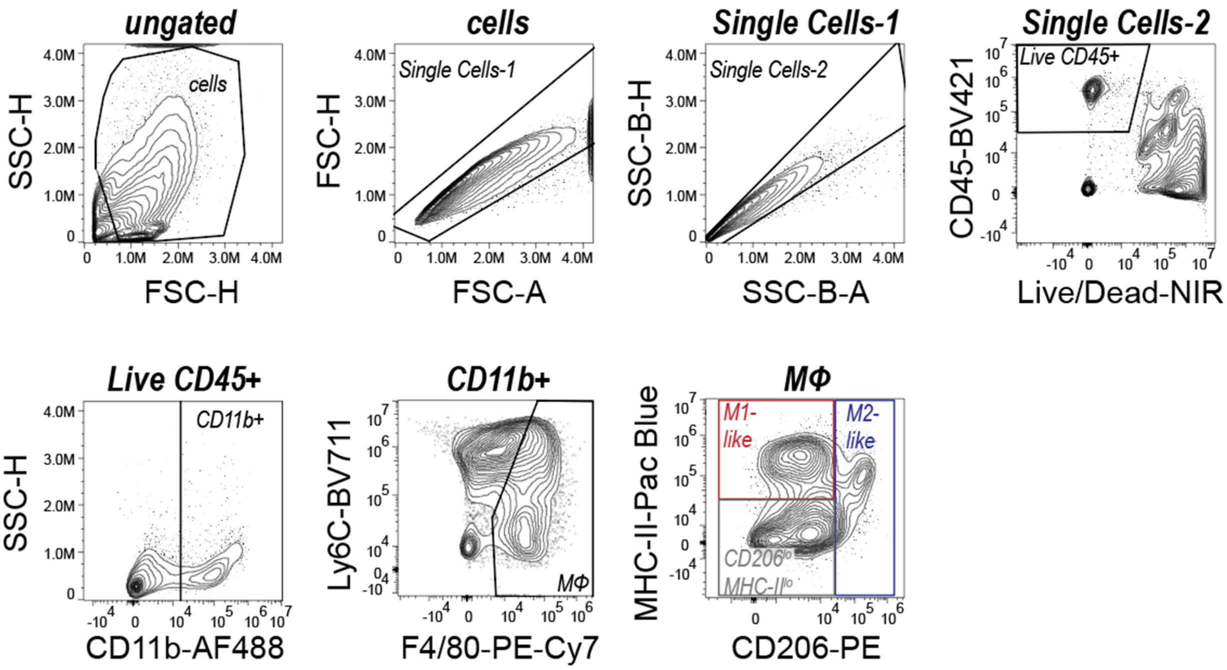

Supplementary Figure 6. **Flow cytometric gating strategy for TAMC subsets**. Shown is the gating strategy used in **Fig. 1i-l** for CD206^{low}MHCII^{low} M0-like macrophages, CD206^{low}MHCII^{high} M1-like macrophages, and CD206^{high}MHCII^{low} M2-like macrophages.

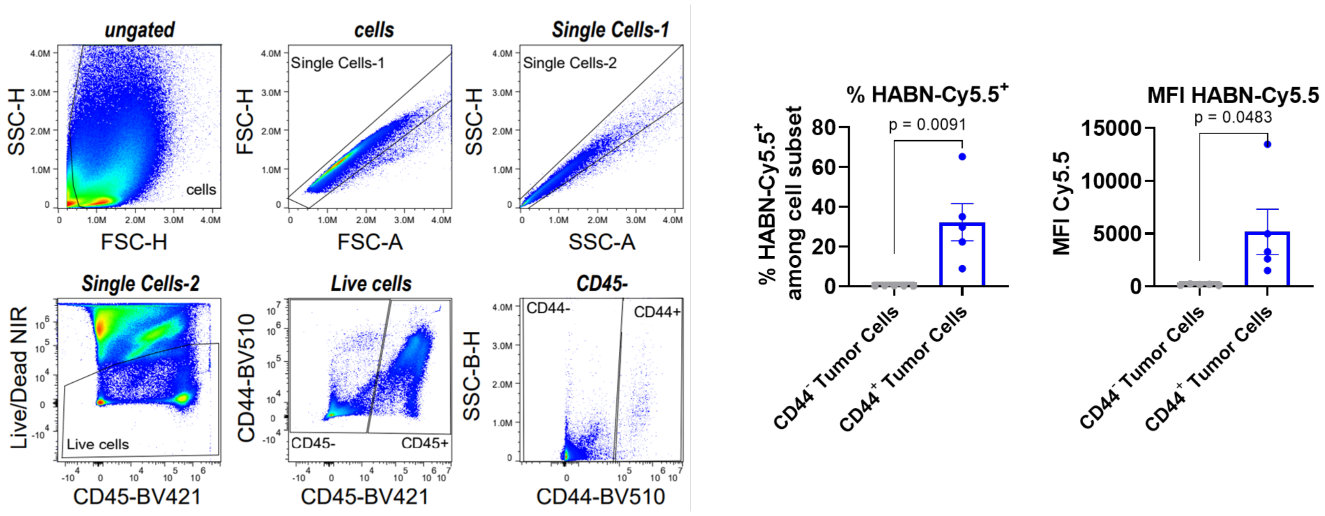

Supplementary Figure 7. **HABN accumulates in CD44^{+} tumor cells *in vivo*.** MC38 tumor-bearing mice were administered IV on day 25 with 10 mg/kg of HABN-Cy5.5 or 0.25 mg/kg of free Cy5.5 (equivalent mass of Cy5.5 in HABN-Cy5.5), followed by flow cytometric analysis of tumor tissues on day 26. Shown is the gating strategy for CD45- tumor cells and quantification of uptake of HABN-Cy5.5 among CD45-CD44+ or CD45-CD44- tumor cells. The data represent mean ± s.e.m., biological replicates with n = 5. *p < 0.05, **p < 0.01, ***p < 0.001, ****p < 0.0001 analyzed by two-sided Student's T-test.

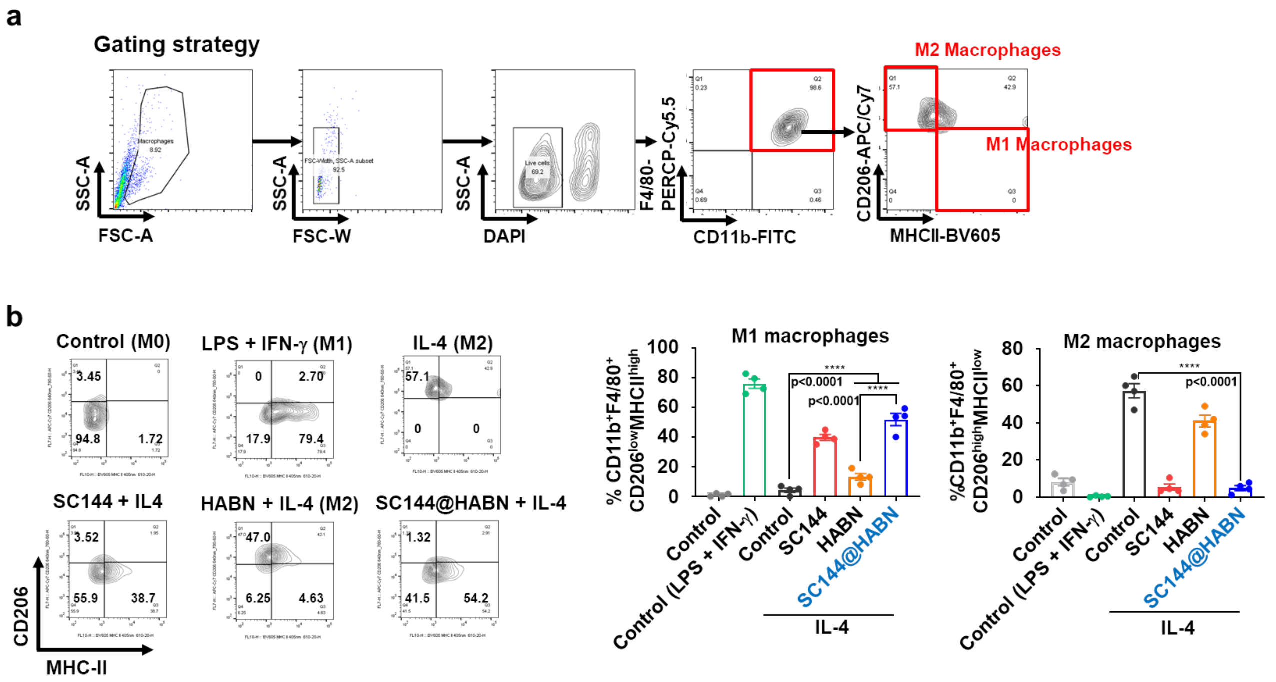

Supplementary Figure 8. **SC144@HABN alters polarization of macrophages *in vitro*.** **a,** Gating strategy for $CD45^{+}CD11b^{+}F4/80^{+}MHC\text{-}II^{+}$ M1- macrophages and $CD45^{+}CD11b^{+}F4/80^{-}CD206^{+}$ M2-macrophages. **b**, Frequencies of $CD45^{+}CD11b^{+}F4/80^{+}MHC\text{-}II^{+}$ M1- macrophages and $CD45^{+}CD11b^{+}F4/80^{-}CD206^{+}$ M2-macrophages after treatment of SC144 (10μM), HABN (40 μg/ml), or SC144@HABN (10 μM of SC144; 40 μg/ml of HABN), or fresh medium for 24 in the presence or absence of IL-4 (20 ng/ml). The data represent mean ± s.e.m. biological replicates with n = 4. **p < 0.01, ****p < 0.0001 analyzed by one-way ANOVA with Tukey's HSD multiple comparison post hoc test.

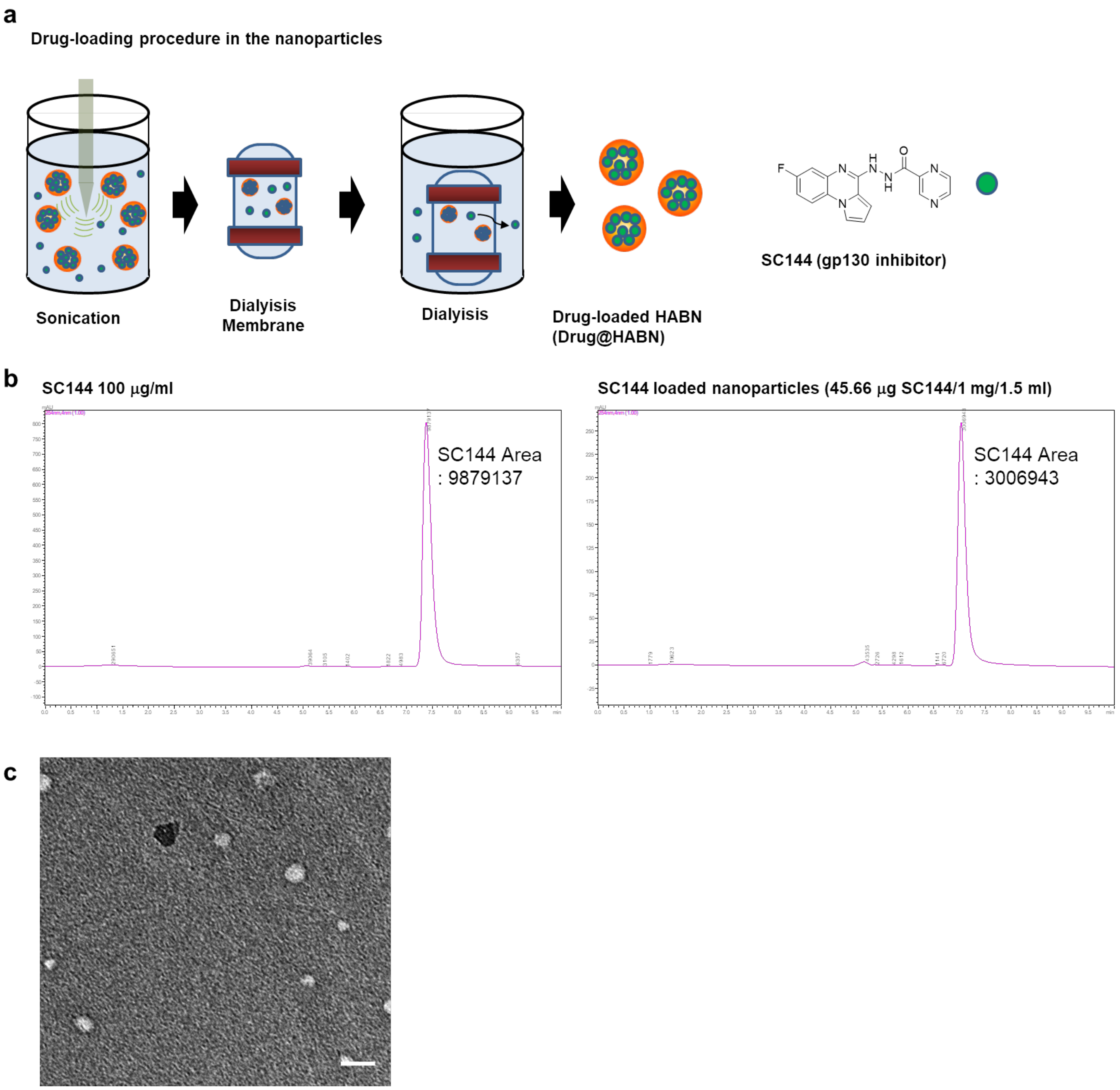

Supplementary Figure 9. **Preparation of SC144-loaded HABN (SC144@HABN). a,** Scheme for the drug loading into HABN. **b,** HPLC chromatograms of SC144. **c,** TEM image of SC144@HABN. Scale bar = 200 nm.

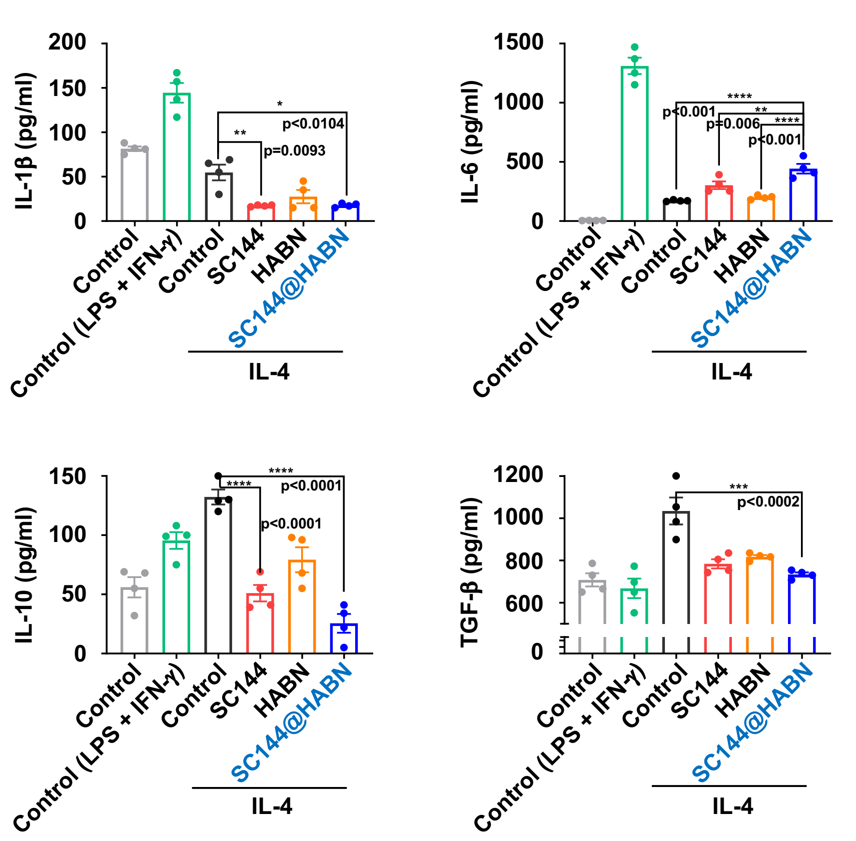

Supplementary Figure 10. **SC144@HABN promotes the secretion of pro-inflammatory cytokines from macrophages while decreasing anti-inflammatory cytokines.** Cytokine levels released from BMDMs treated for 24 h with SC144 (10 μM), HABN (40 μg/ml), or SC144@HABN (10 μM of SC144; 40 μg/ml of HABN), or fresh medium for 24 in the presence or absence of IL-4 (20 ng/ml). The data represent mean ± s.e.m. biological replicates with n = 4. *p < 0.05, **p < 0.01, ***p < 0.001, ****p < 0.0001 analyzed by one-way ANOVA with Tukey's HSD multiple comparison post hoc test.

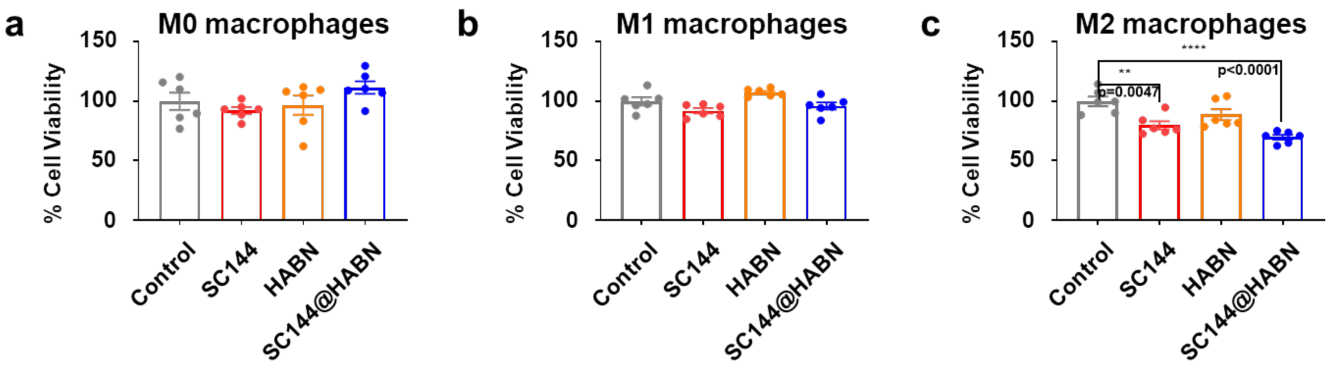

Supplementary Figure 11. **SC144@HABN induces cytotoxicity of M2-like macrophages.** BMDM cells were pre-treated with LPS (100 ng/ml) and IFN-γ (10 ng/ml), IL-4 (20 ng/ml), or control medium, followed by treatment with 10 μM of SC144, 40 μg/ml of HABN, SC144@HABN (10 μM of SC144; 40 μg/ml of HABN), or PBS. After 24 h, cell viability was measured with CCK-8 assay. The data represent mean ± s.e.m., biological replicates with n = 6. **p < 0.01, ***p < 0.001 analyzed by one-way ANOVA with Tukey's HSD multiple comparison post hoc test.

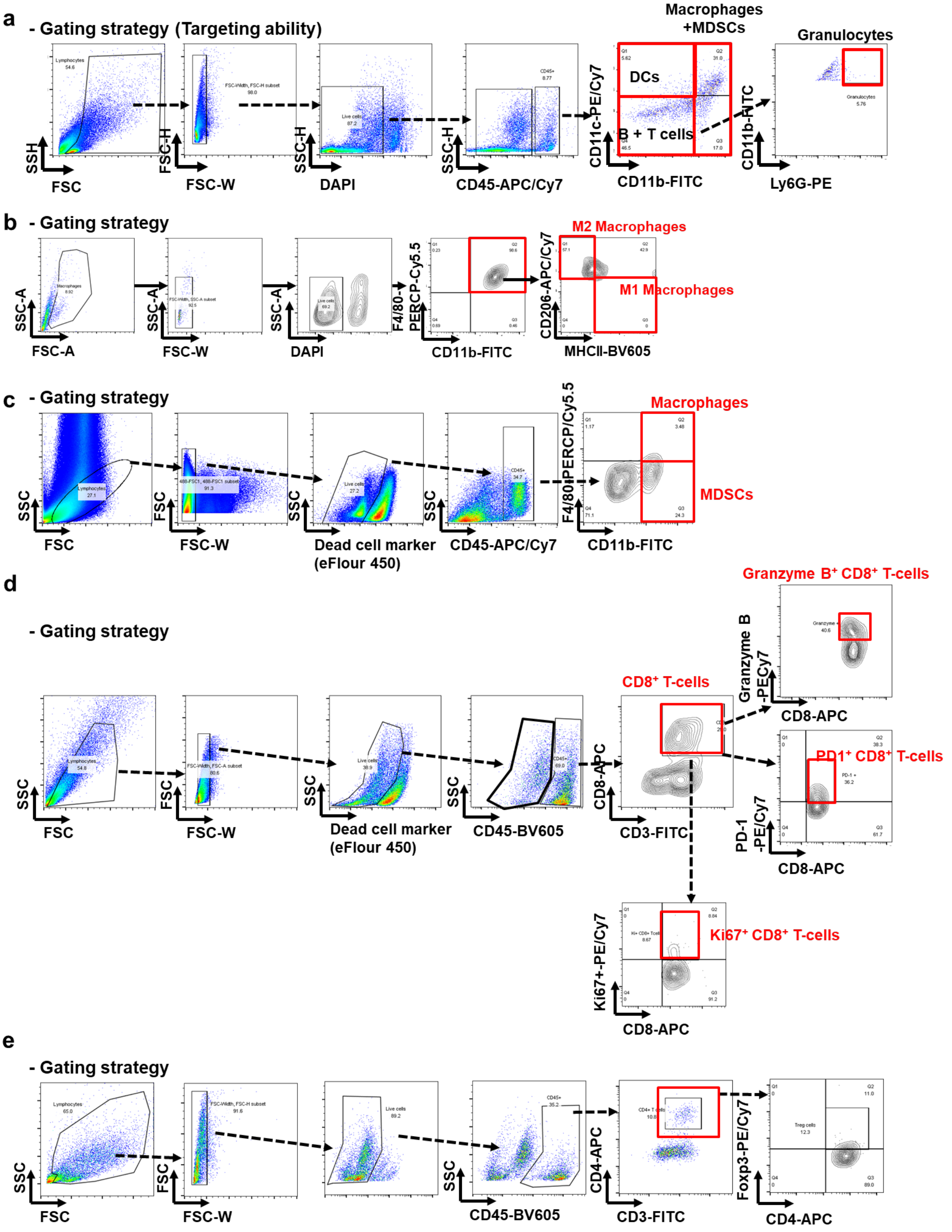

Supplementary Figure 12. Flow cytometric analysis of the tumor microenvironment. a, Gating strategy for immune cells in M38-bearing mice treated with 10 mg/kg of HABN-Cy5.5 (equivalent mass of free Cy5.5), or 0.25 mg/kg of free Cy5.5. **b**, Gating strategy for analyzing CD45$^+$CD11b$^+$F4/80$^+$MHC-II$^+$ M1- macrophages and CD45$^+$CD11b$^+$F4/80$^-$CD206$^+$ M2-macrophages. **c**, Gating strategy for analyzing CD45$^+$CD11b$^+$F4/80$^+$ macrophages, and CD45$^+$CD11b$^+$ F4/80$^-$ MDSCs. **d,** Gating strategy of analyzing CD45$^+$CD3$^+$CD8$^+$ T-cells, CD45$^+$CD3$^+$CD8$^+$Ki67$^+$ T-cells, CD45$^+$CD3$^+$CD8$^+$Granzyme B$^+$ T-cells, CD45$^+$CD3$^+$CD8$^+$PD-1$^+$ T-cells. **e,** Gating strategy for analyzing CD45$^+$CD3$^+$CD4$^+$Foxp3$^+$ T-cells.

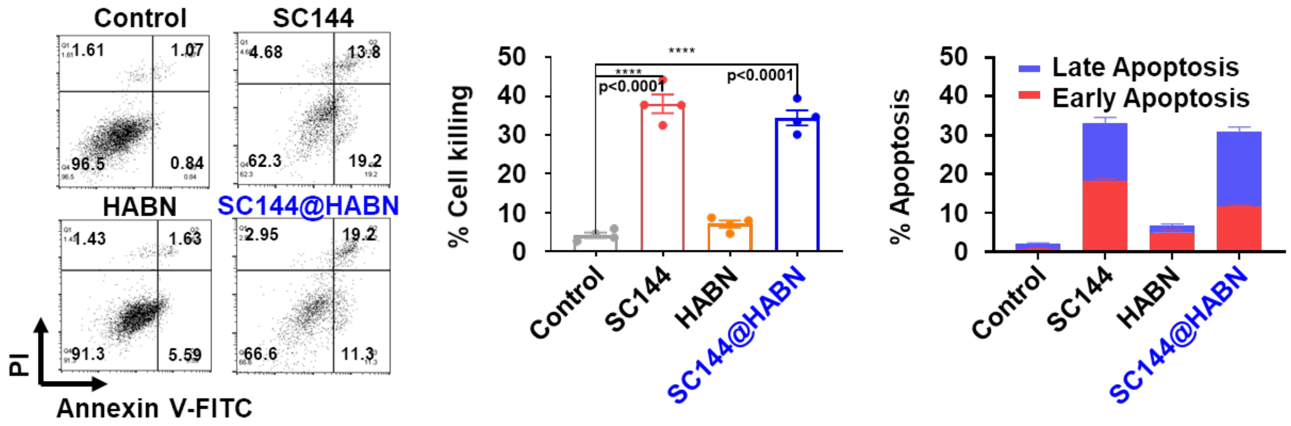

Supplementary Figure 13. **SC144 and SC144@HABN induce apoptosis of MC38 cells *in vitro*.** Flow cytometric analysis of MC38 cells treated with 10 μM of SC144, 40 μg/ml of HABN, SC144@HABN (10 μM of SC144; 40 μg/ml of HABN), or PBS for 24 h, followed by staining with annexin-V-FITC/PI for the quantification of early apoptotic cells (Annexin V-FITC^{+}/PI^{-}), late apoptotic cells (Annexin V-FITC^{+}/PI^{+}), necrotic cells (Annexin V-FITC^{-}/PI^{+}), and viable cells (Annexin V-FITC^{-}/PI^{-}). The data represent mean ± s.e.m., biological replicates with n = 4. ****p < 0.0001 analyzed by one-way ANOVA with Tukey's HSD multiple comparison post hoc test.

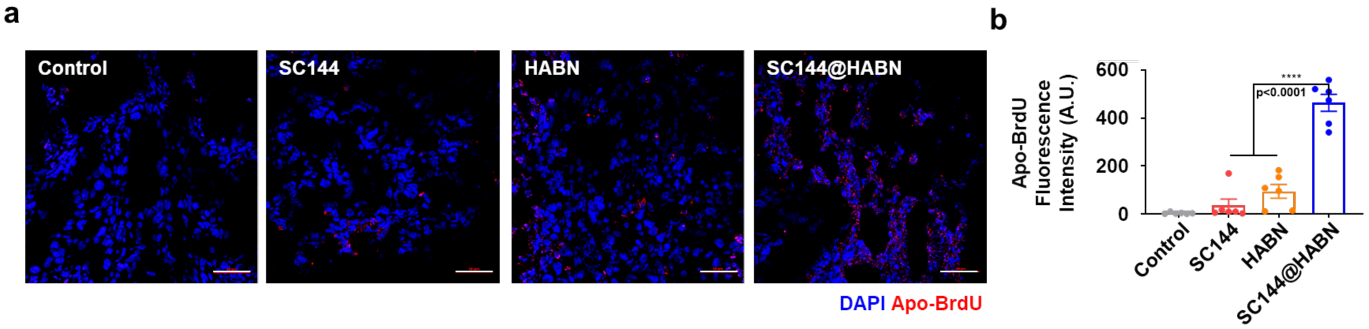

Supplementary Figure 14. SC144@HABN induces apoptosis in MC38 tumor *in vivo*. a-b, MC38 tumor-bearing C57BL/6 mice were administered IV with SC144 (5 mg/kg), HABN (50 mg/kg) SC144@HABN (5 mg/kg of SC144; 50 mg/kg of HABN), or PBS on days 11, 13, and 15. Tumor tissues were excised on day 18, processed by the TUNEL assay, visualized with confocal microscopy, followed by quantification of Apo-BrdU fluorescence signal. Scale bar = 50 μm. Data are presented as mean ± s.e.m., biological replicates with n = 6. ****p < 0.0001 analyzed by one-way ANOVA with Tukey's HSD multiple comparison post hoc test.

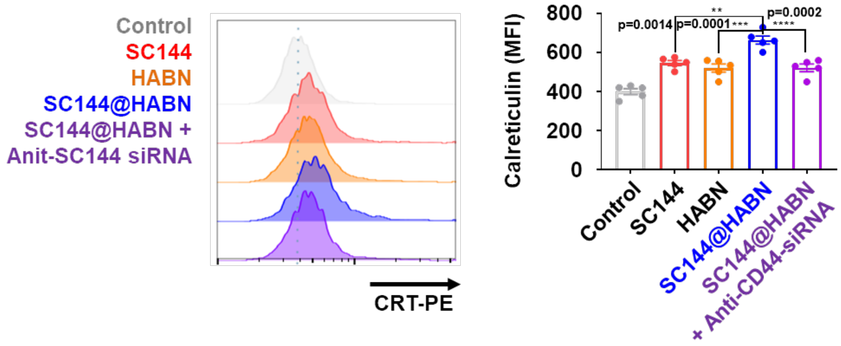

Supplementary Figure 15. **SC144@HABN induces CRT on MC38 cells.** Flow cytometry analysis of MC38 cells incubated *in vitro* with 10 μM of SC144, 40 μg/ml of HABN, SC144@HABN (10 μM of SC144; 40 μg/ml of HABN), or PBS for 24 h and stained with anti-CRT antibody. The data represent mean ± s.e.m., biological replicates with n = 5. ***p < 0.001, ****p < 0.0001 analyzed by one-way ANOVA with Tukey's HSD multiple comparison post hoc test.

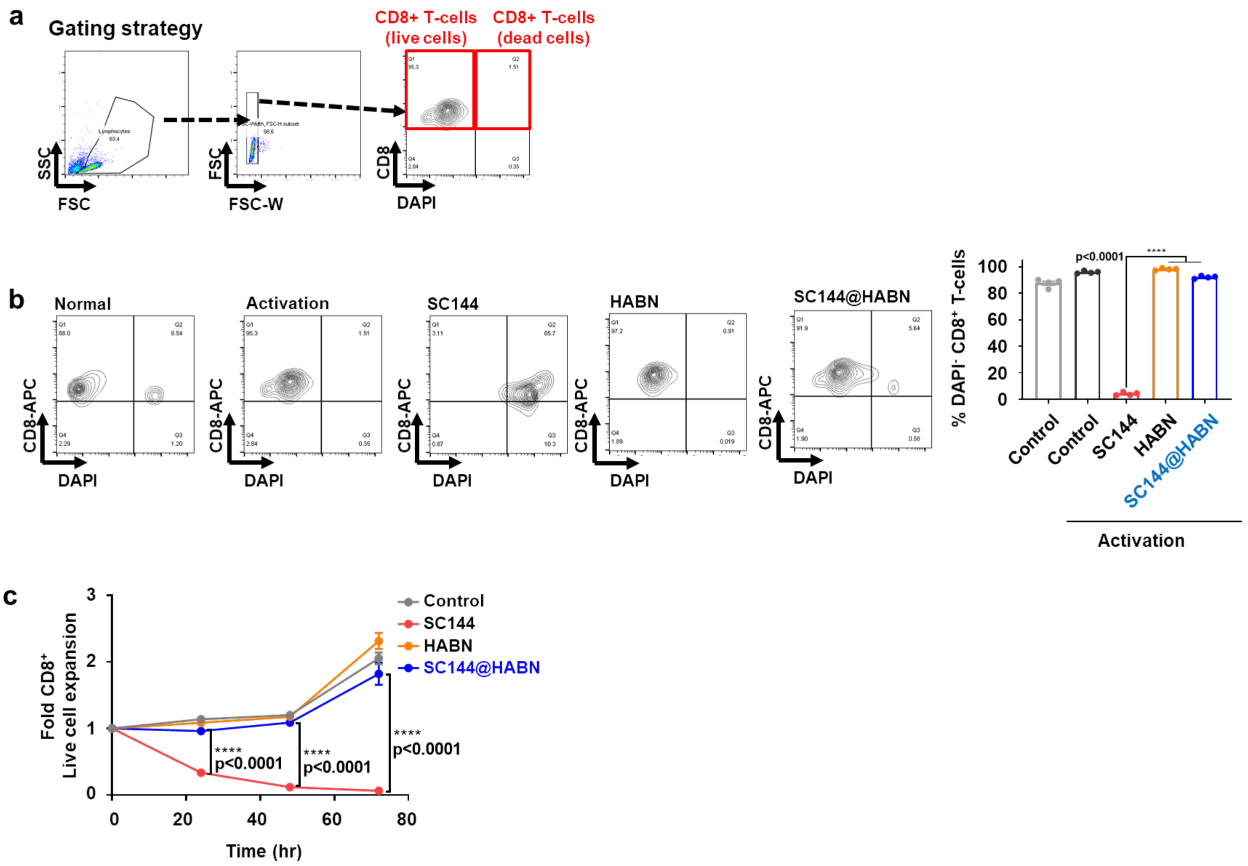

Supplementary Figure 16. HABN reduces the cytotoxicity of SC144 among CD8^{+} T-cells. a-c, CFSE-labeled CD8^{+} T-cells were incubated *in vitro* with SC144 (2 μM), HABN (8 μg/ml), or SC144@HABN (2 μM of SC144; 8 μg/ml of HABN) for 48 hours, followed by flow cytometric analysis for DAPI signal (**b**) among CD8^{+} T-cells. **c**, Expansion of live CD8^{+} T-cells quantified over 48 hr treatment with SC144 (2 μM), HABN (8 μg/ml), or SC144@HABN (2 μM of SC144; 8 μg/ml of HABN). The data represent mean ± s.e.m., biological replicates with n = 5. ****p < 0.0001 analyzed by one-way ANOVA (b) or two-way ANOVA (c), with Tukey's HSD multiple comparison post hoc test.

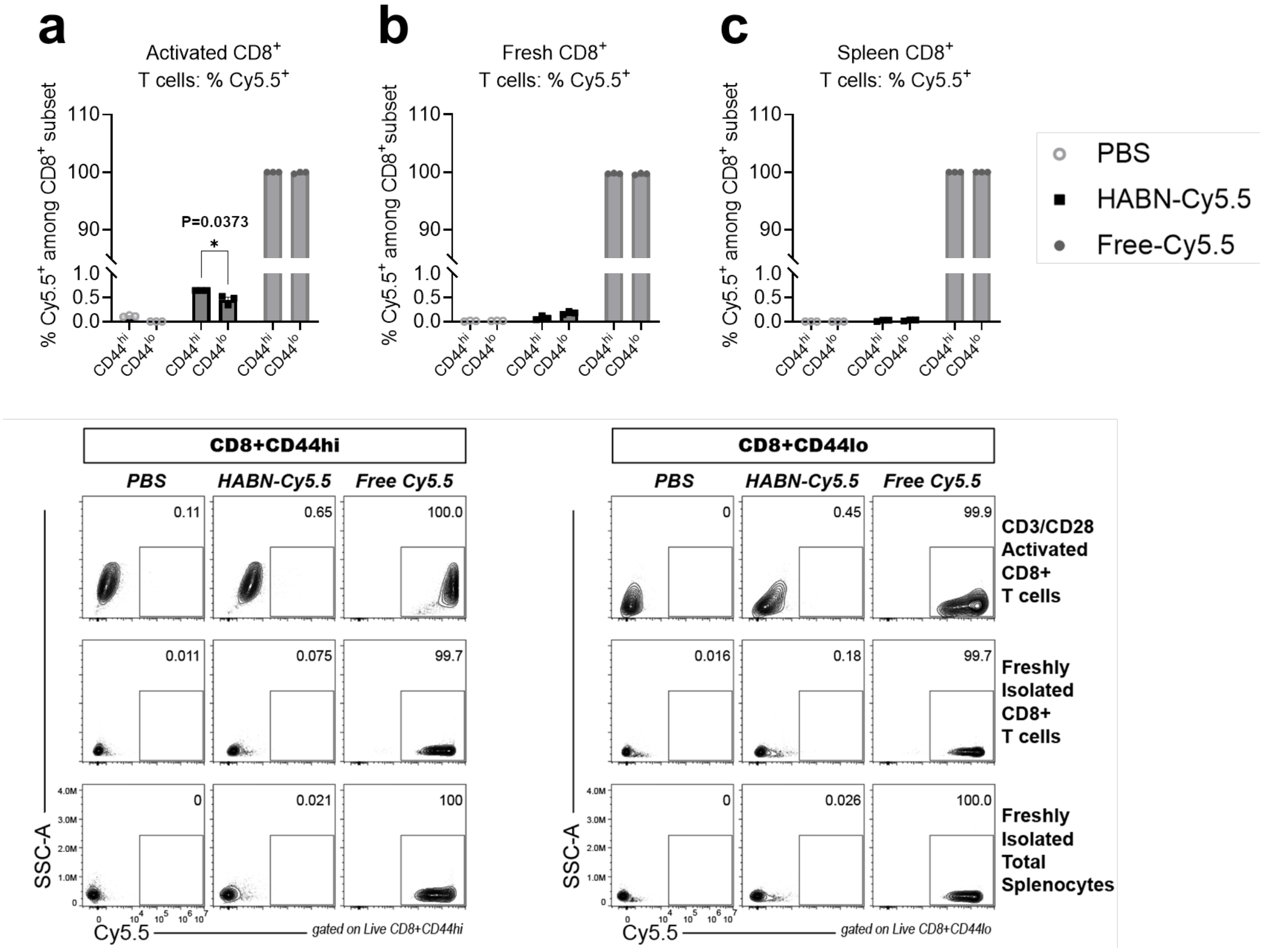

Supplementary Figure 17. HABN reduces the cytotoxicity of SC144 among CD8$^+$ T-cells. a-c, CD8+ T cells were isolated from spleens of naïve C57BL6 mice using StemCell's CD8 Negative Selection Kit. Isolated T cells were activated with plate bound αCD3 (1 μg/mL) plus soluble αCD28 (0.5 μg/mL) and IL-2 (10 ng/mL) for 3 days. On the day of co-culture, spleens from naïve C57BL6 were collected and CD8 T cells were isolated for use as not-activated CD8+ T cell controls. **a,** Freshly isolated, **b,** αCD3/CD28-activated, and **c,** not-activated total splenocytes were co-cultured with PBS, HABN-Cy5.5 (20 μg/mL), or Free-Cy5.5 (0.8 μg/mL) for 1 hour. Cy5.5 uptake among CD44^{hi} vs. CD44^{lo} populations was quantified by flow cytometric analysis. The data represent mean ± s.e.m., biological replicates with n = 3. *$p < 0.05$, analyzed by one-way ANOVA with Tukey's HSD multiple comparison post hoc test.

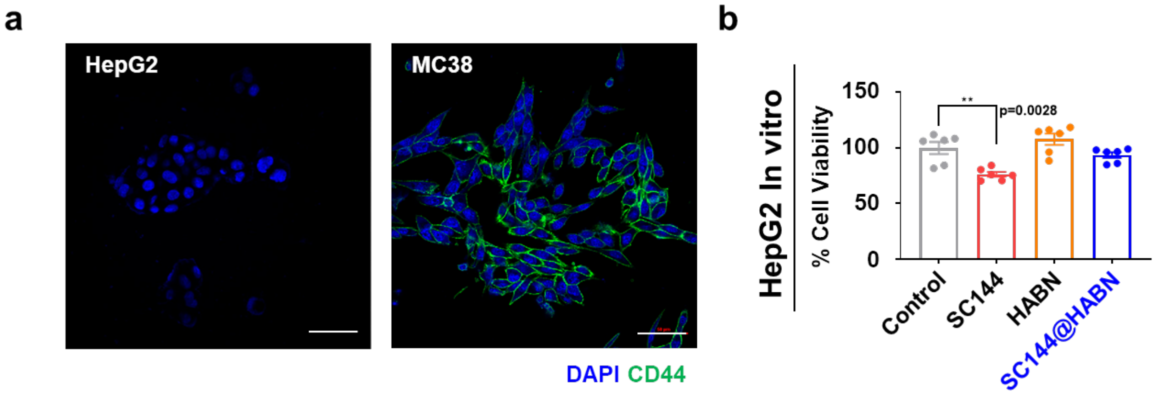

Supplementary Figure 18. SC144@HABN shows no toxicity in HepG2 cells expressing low levels of CD44. a, Confocal microscopy images of HepG2 and MC38 cells stained with anti-CD44 antibody. Scale bars = 50 μm. **b,** HepG2 cells were treated with 10 μM of SC144, 40 μg/ml of HABN, SC144@HABN (10 μM of SC144; 40 μg/ml of HABN), or PBS. After 24 h, cell viability was measured with CCK-8 assay. Data are presented as mean ± s.e.m., biological replicates with n = 6. ***p < 0.001 analyzed by one-way ANOVA with Tukey's HSD multiple comparison post hoc test.

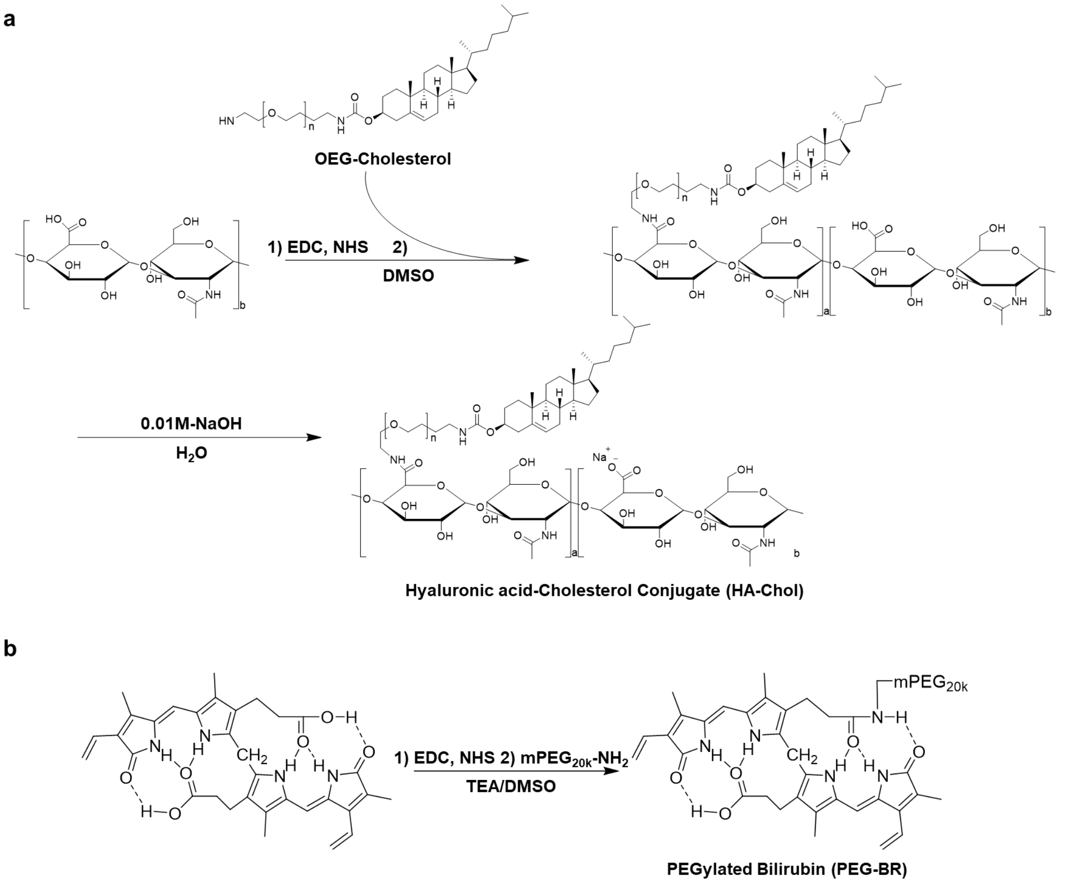

Supplementary Figure 19. A scheme for the synthesis of hyaluronic acid-cholesterol conjugate (HA-Chol, a) and PEGylated bilirubin (PEG-BR, b).

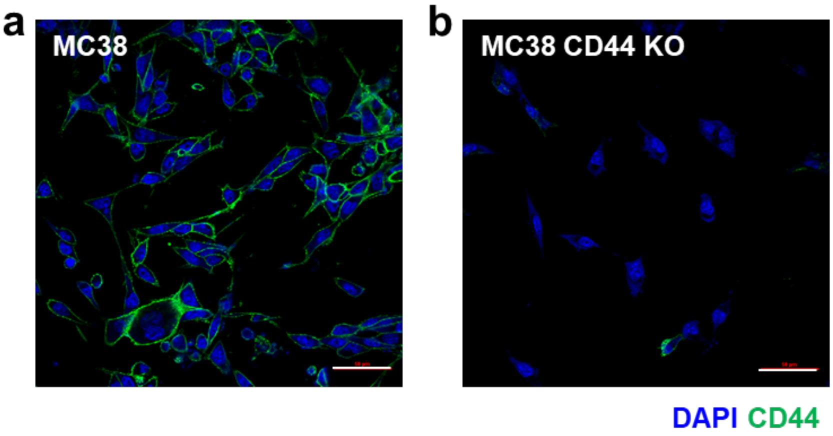

Supplementary Figure 20. CD44 expression on MC38 or CD44-KO MC38 cells. CRISPR/Cas9 system was used to generate CD44 knock-out MC38 cell line. MC38 or CD44-KO MC38 cells were incubated with FITC-conjugated anti-CD44 antibody for 1 h, followed by confocal microscopy. Scale bars = 50 μm.

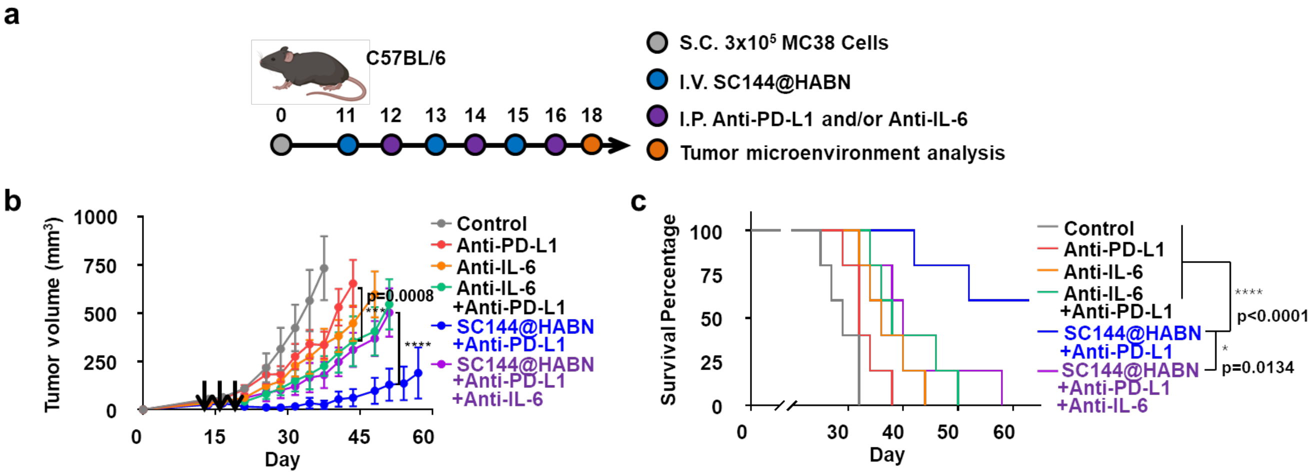

Supplementary Figure 21. IL-6 has a crucial role in the anti-tumor efficacy of SC144@HABN and anti-PD-L1 combo therapy. a-c, C57BL/6 mice bearing MC38 tumor were administered IV with SC144 (5 mg/kg), HABN (50 mg/kg) SC144@HABN (5 mg/kg of SC144; 50 mg/kg of HABN), or PBS on days 11, 13, and day 15 with or without intraperitoneal administration of anti-mouse PD-L1 (5 mg/kg) and/or anti-mouse IL-6 (10 mg/kg) on days 12, 14, and 16. Shown are tumor growth curves (b) and animal survival (c). The data represent mean ± s.e.m., biological replicates with n = 5. *$p < 0.05$, **$p < 0.01$, ***$p < 0.001$, ****$p < 0.0001$ analyzed by two-way ANOVA (b) with Tukey's HSD multiple comparison post hoc test, or Kaplan–Meier survival analysis with the log-rank (Mantel–Cox) test (c). .

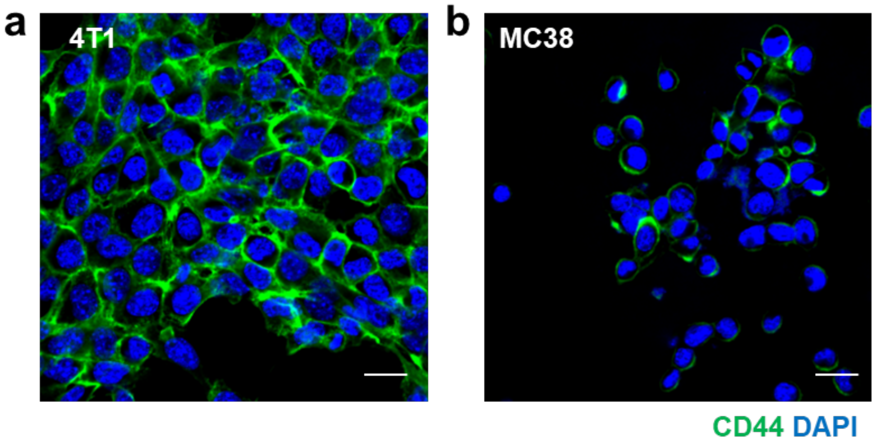

Supplementary Figure 22. CD44 expression on 4T1 and MC38 tumor cells. Confocal microscopy images of 4T1 or MC38 cells incubated with FITC-conjugated anti-CD44 antibody for 1 h. Scale bars = 20 μm.

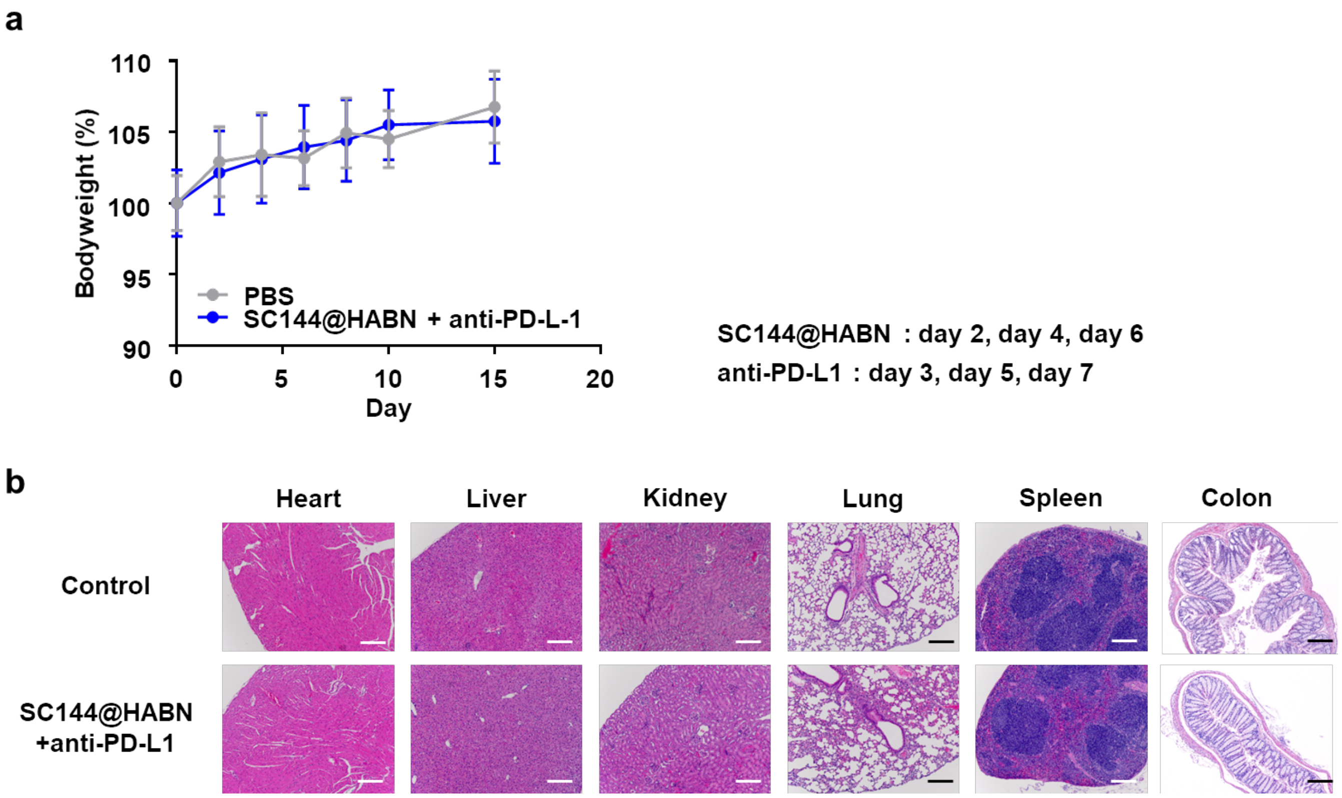

Supplementary Figure 23. Safety profiles of SC144@HABN and anti-PD-L1 combo therapy. A-c, Mice were administered intravenously with SC144@HABN (5 mg/kg of SC144; 50 mg/kg of HABN) or PBS on days 2, 4, and 6 and administered intraperitoneally with 5 mg/kg of anti-PD-L1 antibody on days 3, 5, and 7. **A,** Daily bodyweight changes in each group for 15 days. **B,** Major organ (heart, liver, kidney, lung, spleen, and colon) sections stained with hematoxylin and eosin (H&E) were analyzed for systemic toxicity evaluation. Data are presented as mean ± s.e.m., biological replicates with n = 5. Scale bars = 200 μm.

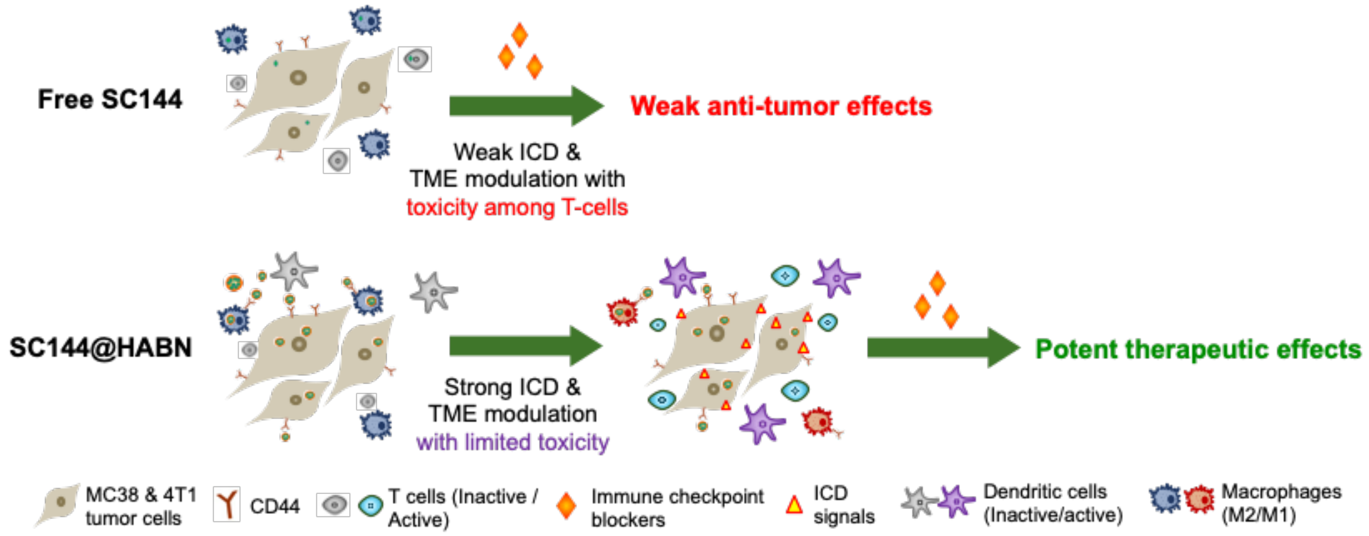

Supplementary Figure 24. HABN-based combination chemoimmunotherapy induces ICD and modulates TME with minimal toxicity, leading to strong anti-tumor effects.

Supplementary Table 1. Antibody Information

Antibodies	Clone Number	Provider	Catalog	Dose or dilution used
Anti-mouse IL-6 Antibody	MP5-20F3	Bioxcell	#BE0046	In vivo treatment (10 mg/kg)
Anti-mouse PD-L1 Antibody	10F.9G2	Bioxcell	#BE0101	In vivo treatment (5 mg/kg)
Anti-mouse CSFIR antibody	AFS98	Bioxcell	#BE0213	In vivo treatment (20 mg/kg)
FITC-Anti-mouse CD3 Antibody	17A2	Biolegend	#100203	1/100 dilution
FITC-Anti-mouse CDllb antibody	MI/70	Biolegend	#101205	1/100 dilution
PE-Cy6-Anti-mouse CDllc Antibody	MI/70	Biolegend	#117317	1/100 dilution
PE-Anti-mouse Ly6G Antibody	1A8	Biolegend	#127607	1/100 dilution
BV650-Anti-mouse MHCII Antibody	M5/114.15.2	Biolegend	#100545	1/100 dilution
APC-Cy7-Anti-mouse CD45 Antibody	30- FII	Biolegend	#103115	1/100 dilution
BV605-Anti-mouse CD45 Antibody	30-FII	Biolegend	#103139	1/100 dilution
APC-Cy7-Anti-mouse CD206 Antibody	C068C2	Biolegend	#141719	1/100 dilution
PERCP-Cy5.5-Anti-mouse F4/80 Antibody	BM8	Biolegend	#123127	1/100 dilution
PE-Cy7-Anti-mouse PD-1 Antibody	RMPl-30	Biolegend	#109109	1/100 dilution
PE-Anti-mouse PD-L1 Antibody	10F.9G2	Biolegend	#124307	1/100 dilution
PE-Cy7-Anti-mouse Ki67 Antibody	16A8	Biolegend	#652425	1/100 dilution

APC-Anti-mouse CD4 Antibody	RM4-5	eBioscience	#17-0042-82	1/100 dilution
PE-Cy7-Anti-mouse Granzyme B Antibody	NGZB	eBioscience	#25-8898-82	1/100 dilution
FITC-Anti-mouse CD44 Antibody	IM7	eBioscience	#11-0441-82	1/100 dilution
PE-Cy7-Anti-mouse FOXP3 Antibody	FJK-16S	eBioscience	#25-5773-82	1/100 dilution
APC-Anti-mouse CD8 Antibody	53-6.7	BD bioscience	#553035	1/100 dilution
PE-Anti-mouse CRT Antibody	FMC 75	Abcam	ab83220	1/100 dilution
Antimouse CD16/32 Antibody	93	eBioscience	#14-0161-82	1/20 dilution
CD45-BV421	30-FlI	Biolegend	#103133	1/200 dilution
MHC-II-Pacific Blue	M5/114.15.2	Biolegend	#107619	1/200 dilution
CD44-BV510	IM7	Biolegend	#103039	1/200 dilution
Ly6C-BV711	HKl.4	Biolegend	#128037	1/200 dilution
CDllb-FITC	Ml/70	Biolegend	#101205	1/200 dilution
CD206-PE	C068C2	Biolegend	#141705	1/200 dilution
F4/80-PE-Cy7	BM8	Biolegend	#123113	1/200 dilution
CD8-Pacific Blue	53-6.7	Biolegend	#100728	1/200 dilution
CD44- PE-Dazzle	IM7	Biolegend	#103055	1/200 dilution
