# Supplementary material for: Hyaluronic acid-bilirubin nanomedicine-based combination chemoimmunotherapy
Source: Nat Commun. 2023 Aug 8;14:4771. doi: 10.1038/s41467-023-40270-5 (PMC10409794; doi:10.1038/s41467-023-40270-5)
Supplement: Supplementary file 2 — Reporting Summary [file 41467_2023_40270_MOESM2_ESM.pdf]

nature portfolio

Corresponding author(s): Yonghyun Lee and James Moon

Last updated by author(s): Jul 14, 2023

Reporting Summary

Nature Portfolio wishes to improve the reproducibility of the work that we publish. This form provides structure for consistency and transparency in reporting. For further information on Nature Portfolio policies, see our Editorial Policies and the Editorial Policy Checklist.

Statistics

For all statistical analyses, confirm that the following items are present in the figure legend, table legend, main text, or Methods section.

n/a	Confirmed	
☐	☒	The exact sample size (n) for each experimental group/condition, given as a discrete number and unit of measurement
☐	☒	A statement on whether measurements were taken from distinct samples or whether the same sample was measured repeatedly
☐	☒	The statistical test(s) used AND whether they are one- or two-sided<br>*Only common tests should be described solely by name; describe more complex techniques in the Methods section.*
☐	☒	A description of all covariates tested
☐	☒	A description of any assumptions or corrections, such as tests of normality and adjustment for multiple comparisons
☐	☒	A full description of the statistical parameters including central tendency (e.g. means) or other basic estimates (e.g. regression coefficient) AND variation (e.g. standard deviation) or associated estimates of uncertainty (e.g. confidence intervals)
☐	☒	For null hypothesis testing, the test statistic (e.g. F, t, r) with confidence intervals, effect sizes, degrees of freedom and P value noted<br>*Give P values as exact values whenever suitable.*
☒	☐	For Bayesian analysis, information on the choice of priors and Markov chain Monte Carlo settings
☐	☒	For hierarchical and complex designs, identification of the appropriate level for tests and full reporting of outcomes
☐	☒	Estimates of effect sizes (e.g. Cohen's d, Pearson's r), indicating how they were calculated

Our web collection on statistics for biologists contains articles on many of the points above.

Software and code

Policy information about availability of computer code

Data collection	TEM images were acquired by AMT602 software. Histology images were acquired using Matra Quantitative Pathology Workstation (v.1.0.3). Confocal fluorescence images were acquired by Nikon A1 NIS elements Imaging software (v.5.02). Flow cytometric data were collected using a Beckman CytoFLEX Cell Analyzer with CytExpert Software (v 2.2.0.97) and a Cytek Aurora Analyzer with SpectroFlo software (v 3.0). In vivo images were acquired using IVIS Lumina Living Image Software(v.4.5.5). DLS analysis was collected in Zetasizer software version 7.13.
Data analysis	Histology images were analyzed by inForm image analysis software (v.2.3.0). Confocal fluorescence images were analyzed by Nikon A1 NIS elements Imaging software (v.5.02). Flow cytometry analysis was done using FlowJo (v.10.5) (Tree Star). In vivo images were analyzed using IVIS Lumina Living Image (v.4.5.5) Software. Statistical analysis was done in GraphPad Prism 8.0. DLS analysis was done in Zetasizer software version 7.13.

For manuscripts utilizing custom algorithms or software that are central to the research but not yet described in published literature, software must be made available to editors and reviewers. We strongly encourage code deposition in a community repository (e.g. GitHub). See the Nature Portfolio guidelines for submitting code & software for further information.

Data

Policy information about availability of data

All manuscripts must include a data availability statement. This statement should provide the following information, where applicable:

- Accession codes, unique identifiers, or web links for publicly available datasets
- A description of any restrictions on data availability
- For clinical datasets or third party data, please ensure that the statement adheres to our policy

The authors declare that data supporting the findings of this study are available within the article and its Supplementary Information files. All relevant data can be provided by the authors upon reasonable request.

Research involving human participants, their data, or biological material

Policy information about studies with human participants or human data. See also policy information about sex, gender (identity/presentation), and sexual orientation and race, ethnicity and racism.

Reporting on sex and gender	*Use the terms sex (biological attribute) and gender (shaped by social and cultural circumstances) carefully in order to avoid confusing both terms. Indicate if findings apply to only one sex or gender; describe whether sex and gender were considered in study design; whether sex and/or gender was determined based on self-reporting or assigned and methods used. Provide in the source data disaggregated sex and gender data, where this information has been collected, and if consent has been obtained for sharing of individual-level data; provide overall numbers in this Reporting Summary. Please state if this information has not been collected. Report sex- and gender-based analyses where performed, justify reasons for lack of sex- and gender-based analysis.*
Reporting on race, ethnicity, or other socially relevant groupings	*Please specify the socially constructed or socially relevant categorization variable(s) used in your manuscript and explain why they were used. Please note that such variables should not be used as proxies for other socially constructed/relevant variables (for example, race or ethnicity should not be used as a proxy for socioeconomic status). Provide clear definitions of the relevant terms used, how they were provided (by the participants/respondents, the researchers, or third parties), and the method(s) used to classify people into the different categories (e.g. self-report, census or administrative data, social media data, etc.) Please provide details about how you controlled for confounding variables in your analyses.*
Population characteristics	*Describe the covariate-relevant population characteristics of the human research participants (e.g. age, genotypic information, past and current diagnosis and treatment categories). If you filled out the behavioural & social sciences study design questions and have nothing to add here, write "See above."*
Recruitment	*Describe how participants were recruited. Outline any potential self-selection bias or other biases that may be present and how these are likely to impact results.*
Ethics oversight	*Identify the organization(s) that approved the study protocol.*

Note that full information on the approval of the study protocol must also be provided in the manuscript.

Field-specific reporting

Please select the one below that is the best fit for your research. If you are not sure, read the appropriate sections before making your selection.

[x] Life sciences [ ] Behavioural & social sciences [ ] Ecological, evolutionary & environmental sciences

For a reference copy of the document with all sections, see nature.com/documents/nr-reporting-summary-flat.pdf

Life sciences study design

All studies must disclose on these points even when the disclosure is negative.

Sample size	Sample sizes were chosen based on our preliminary data from at least two pilot experiments and previously published results in the literature.
Data exclusions	No data were excluded.
Replication	All experiments were repeated at least twice with similar results.
Randomization	Mice were assigned randomly to experimental groups.
Blinding	The investigators were not blinded to allocation during experiments and outcome assessment since our data analyses are based on objectively measurable data.

Reporting for specific materials, systems and methods

We require information from authors about some types of materials, experimental systems and methods used in many studies. Here, indicate whether each material, system or method listed is relevant to your study. If you are not sure if a list item applies to your research, read the appropriate section before selecting a response.

Materials & experimental systems

n/a	Involved in the study
☐	☒ Antibodies
☐	☒ Eukaryotic cell lines
☒	☐ Palaeontology and archaeology
☐	☒ Animals and other organisms
☒	☐ Clinical data
☒	☐ Dual use research of concern
☒	☐ Plants

Methods

n/a	Involved in the study
☒	☐ ChIP-seq
☐	☒ Flow cytometry
☒	☐ MRI-based neuroimaging

Antibodies

Antibodies used

Anti-mouse IL-6 Antibody (#BE0046, Clone: MP5-20F3, Bioxcell, 10 mg/kg), Anti-mouse PD-L1 Antibody (#BE0101, Clone: 10F.9G2, Bioxcell, 5 mg/kg), Anti-mouse CSFIR antibody, (#BE0213, Clone: AFS98, Bioxcell, 20 mg/kg), FITC-Anti-mouse CD3 Antibody (1/100, #100203, Clone: 17A2, Biolegend), FITC-Anti-mouse CDllb antibody (1/100, #101205, Clone: Ml/70, Biolegend), PE-Cy6-Anti-mouse CDllc Antibody (1/100, #117317, Clone: N418, Biolegend), PE-Anti-mouse Ly6G Antibody (1/100, #127607, Clone: 1A8, Biolegend), BV650-Anti-mouse MHCII Antibody (1/100, #100545, Clone: M5/114.15.2, Biolegend), APC-Cy7-Anti-mouse CD45 Antibody (1/100, #103115, Clone: 30- Fll, Biolegend), BV605-Anti-mouse CD45 Antibody (1/100, #103139, Clone: 30-Fll, Biolegend), APC-Cy7-Anti-mouse CD206 Antibody (1/100, #141719, Clone: C068C2, Biolegend), PERCP-Cy5.5-Anti-mouse F4/80 Antibody (1/100, #123127, Clone: BM8, Biolegend), PE-Cy7-Anti-mouse PD-1 Antibody (1/100, #109109, Clone: RMPl-30, Biolegend), PE-Anti-mouse PD-L1 Antibody (1/100, #124307, Clone: 10F.9G2, Biolegend), PE-Cy7-Anti-mouse Ki67 Antibody (1/100, #652425, Clone: 16A8, Biolegend). APC-Anti-mouse CD4 Antibody (1/100, #17-0042-82, Clone: RM4-5, eBioscience), PE-Cy7-Anti-mouse Granzyme B Antibody (1/100, #25-8898-82, Clone: NGZB, ebioscience), FITC-Anti-mouse CD44 Antibody (1/100, #11-0441-82, Clone: IM7, eBioscience), PE-Cy7-Anti-mouse FOXP3 Antibody (1/100, #25-5773-82, Clone: FJK-16S, ebioscience), APC-Anti-mouse CD8 Antibody (1/100, #553035, Clone: 53-6.7, BD bioscience) PE-Anti-mouse CRT Antibody (1/100, ab83220, FMC 75, abcam), Anti-mouse CD16/32 Antibody (1/20, #14-0161-82, Clone: 93, eBioscience), CD45-BV421 (1/200, #103133, Clone: 30-Fll, Biolegend), MHC-II-Pacific Blue (1/200, #107619, Clone: M5/114.15.2, Biolegend), CD44-BV510 (1/200, #103039, Clone: IM7, Biolegend), Ly6C-BV711 (1/200, #128037, Clone: HKl.4, Biolegend), CDllb-FITC (1/200, #101205, Clone: Ml/70, Biolegend), CD206-PE (1/200, #141705, Clone: C068C2, Biolegend), F4/80-PE-Cy7 (1/200, #123113, Clone: BM8, Biolegend), CD8-Pacific Blue (1/200, #100728, Clone: 53-6.7, Biolegend), and CD44-PE-Dazzle (1/200, #103055, Clone: IM7, Biolegend).

Validation

The species and application of the following antibodies were validated by the manufacturer.
1) Anti-mouse IL-6 Antibody, Bioxcell, https://bioxcell.com/invivomab-anti-mouse-il-6-be0046
2) Anti-mouse PD-L1 Antibody, Bioxcell, https://bioxcell.com/invivomab-anti-mouse-pd-l1-b7-h1-be0101
3) Anti-mouse CSFIR antibody, Bioxcell, https://bioxcell.com/invivomab-anti-mouse-csf1r-cd115-be0213?gad=1&gclid=EAIaIQobChMI1tDrzdWL_wIVG8KWCh3_7QqyEAAYASAAEgI9BvD_BwE
4) FITC-Anti-mouse CD3 Antibody, Biolegend, https://www.biolegend.com/en-us/products/fitc-anti-mouse-cd3-antibody-45?GroupID=BLG6732
5) FITC-Anti-mouse CDllb antibody, Biolegend, https://www.biolegend.com/en-us/products/fitc-anti-mouse-human-cd11b-antibody-347?GroupID=BLG10660
6) PE-Cy6-Anti-mouse CDllc Antibody, Biolegend, https://www.biolegend.com/en-us/products/pe-cyanine7-anti-mouse-cd11c-antibody-3086?GroupID=BLG11937
7) PE-Anti-mouse Ly6G Antibody, Biolegend, https://www.biolegend.com/en-us/products/pe-anti-mouse-ly-6g-antibody-4777?GroupID=BLG5803
8) BV650-Anti-mouse MHCII Antibody, Biolegend, https://www.biolegend.com/de-at/products/brilliant-violet-650-anti-mouse-cd4-antibody-7634
9) APC-Cy7-Anti-mouse CD45 Antibody, Biolegend, https://www.biolegend.com/en-us/products/apc-cyanine7-anti-mouse-cd45-antibody-2530?GroupID=BLG1932
10) BV605-Anti-mouse CD45 Antibody, Biolegend, https://www.biolegend.com/en-us/products/brilliant-violet-605-anti-mouse-cd45-antibody-8721?GroupID=BLG6831
11) APC-Cy7-Anti-mouse CD206 Antibody, Biolegend, https://www.biolegend.com/de-de/products/pe-cyanine7-anti-mouse-cd206-mmr-antibody-8631
12) PERCP-Cy5.5-Anti-mouse F4/80 Antibody, Biolegend, https://www.biolegend.com/fr-fr/products/percp-cyanine5-5-anti-mouse-f480-antibody-4303
13) PE-Cy7-Anti-mouse PD-1 Antibody, Biolegend, https://www.biolegend.com/en-us/clone-search/pe-cyanine7-anti-mouse-cd279-pd-1-antibody-3612?GroupID=BLG4702
14) PE-Anti-mouse PD-L1 Antibody, Biolegend, https://www.biolegend.com/en-us/products/pe-anti-mouse-cd274-b7-h1-pd-l1-antibody-4497?GroupID=BLG5396
15) PE-Cy7-Anti-mouse Ki67 Antibody, Biolegend, https://www.biolegend.com/fr-lu/products/pe-cyanine7-anti-mouse-ki-67-antibody-13821
16) APC-Anti-mouse CD4 Antibody, eBioscience, https://www.thermofisher.com/antibody/product/CD4-Antibody-clone-RM4-5-

Monoclonal/17-0042-82
17) PE-Cy7-Anti-mouse Granzyme B Antibody, eBioscience, https://www.thermofisher.com/antibody/product/Granzyme-B-Antibody-clone-NGZB-Monoclonal/25-8898-82
18) FITC-Anti-mouse CD44 Antibody, eBioscience, https://www.thermofisher.com/antibody/product/CD44-Antibody-clone-IM7-Monoclonal/11-0441-82
19) PE-Cy7-Anti-mouse FOXP3 Antibody, eBioscience, https://www.thermofisher.com/antibody/product/FOXP3-Antibody-clone-FJK-16s-Monoclonal/25-5773-82
20) APC-Anti-mouse CD8 Antibody, BD bioscience, https://www.bdbiosciences.com/ko-kr/products/reagents/flow-cytometry-reagents/research-reagents/single-color-antibodies-ruo/apc-rat-anti-mouse-cd8a.553035
21) PE-Anti-mouse CRT Antibody, abcam, https://www.abcam.com/products/primary-antibodies/pe-calreticulin-antibody-fmc-75-ab83220.html
22) Anti- mouse CD16/32 Antibody, eBioscience, https://www.thermofisher.com/antibody/product/CD16-CD32-Antibody-clone-93-Monoclonal/14-0161-82
23) Anti-mouse CD45-BV421, Biolegend, https://www.biolegend.com/en-us/clone-search/brilliant-violet-421-anti-mouse-cd45-antibody-7253?GroupID=BLG6837
24) Anti-mouse MHC-11-Pacific Blue, Biolegend, https://www.biolegend.com/nl-nl/products/pacific-blue-anti-mouse-i-a-i-e-antibody-3136
25) Anti-mouse CD44-BV510, Biolegend, https://www.biolegend.com/en-us/products/brilliant-violet-421-anti-mouse-human-cd44-antibody-7225?GroupID=BLG5925
26) Anti-mouse Ly6C-BV711, Biolegend, https://www.biolegend.com/ja-jp/products/brilliant-violet-711-anti-mouse-ly-6c-antibody-8935
27) Anti-mouse CDllb-FITC, Biolegend, https://www.biolegend.com/en-us/products/fitc-anti-mouse-human-cd11b-antibody-347?GroupID=BLG10660
28) Anti-mouse CD206-PE, Biolegend, https://www.biolegend.com/en-us/products/pe-anti-mouse-cd206-mmr-antibody-7424?GroupID=BLG9506
29) Anti-mouse F4/80-PE-Cy7, Biolegend, https://www.biolegend.com/en-us/products/pe-cyanine7-anti-mouse-f4-80-antibody-4070?GroupID=BLG5319
30) Anti-mouse CDS-Pacific Blue, Biolegend, https://www.biolegend.com/ja-jp/products/pacific-blue-anti-mouse-cd8a-antibody-2856
31) Anti-mouse CD44- PE-Dazzle, Biolegend, https://www.biolegend.com/ja-jp/products/pe-dazzle-594-anti-mouse-human-cd44-antibody-10187

Eukaryotic cell lines

Policy information about cell lines and Sex and Gender in Research

Cell line source(s)	CT26, MC38, and 4T1 cell lines were obtained from the American Type Culture Collection (ATCC).
Authentication	The original cell lines used in this study were authenticated by ATCC, based on analysis of genome sequences such as short tandem repeats. The MC38 CD44 knockout cell line was validated using confocal microscopy (Nikon A1R).
Mycoplasma contamination	All cell lines were tested negative for mycoplasma contamination.
Commonly misidentified lines (See ICLAC register)	No commonly misidentified cell lines were used.

Animals and other research organisms

Policy information about studies involving animals; ARRIVE guidelines recommended for reporting animal research, and Sex and Gender in Research

Laboratory animals	For in vivo studies, 6-8-week-old femal BALB/c mice (Jackson Laboratory, ME) and C57-BL/6 mice (Jackson Laboratory, ME; Laonbio, South Korea) were used.
Wild animals	No wild animals were used.
Reporting on sex	*Indicate if findings apply to only one sex; describe whether sex was considered in study design, methods used for assigning sex. Provide data disaggregated for sex where this information has been collected in the source data as appropriate; provide overall numbers in this Reporting Summary. Please state if this information has not been collected. Report sex-based analyses where performed, justify reasons for lack of sex-based analysis.*
Field-collected samples	No field-collected samples were used.
Ethics oversight	All work performed on animals was in accordance with and approved by the Institutional Animal Care & Use Committee (IACUC) at University of Michigan, Ann Arbor and Ewha Womans University (EWHA IACUC 21-068-4)

Note that full information on the approval of the study protocol must also be provided in the manuscript.

Flow Cytometry

Plots

Confirm that:

- [x] The axis labels state the marker and fluorochrome used (e.g. CD4-FITC).
- [x] The axis scales are clearly visible. Include numbers along axes only for bottom left plot of group (a 'group' is an analysis of identical markers).
- [x] All plots are contour plots with outliers or pseudocolor plots.
- [x] A numerical value for number of cells or percentage (with statistics) is provided.

Methodology

Sample preparation	The sample preparation was described in the Methods.
Instrument	CytoFLEX Cell Analyzer and Cytek Aurora
Software	CytExpert and SpectroFlo were used for collection. CytExpert and FlowJo were used for analysis.
Cell population abundance	Data on the abundance of relevant cell populations are provided in the manuscript.
Gating strategy	Cells were gated first by morphology to exclude cell debris, doublets were then gated out by FSC-A/FSC-W, followed by exclusion of dead cells by gating on dye negative cells.

- [x] Tick this box to confirm that a figure exemplifying the gating strategy is provided in the Supplementary Information.
